# Anticipation and Choice Heuristics in the Dynamic Consumption of Pain Relief

**DOI:** 10.1371/journal.pcbi.1004030

**Published:** 2015-03-20

**Authors:** Giles W. Story, Ivo Vlaev, Peter Dayan, Ben Seymour, Ara Darzi, Raymond J. Dolan

**Affiliations:** 1 Centre for Health Policy, Institute of Global Health Innovation, Imperial College London, London, United Kingdom; 2 Wellcome Trust Centre for Neuroimaging, University College London, London, United Kingdom; 3 Warwick Business School, University of Warwick, Coventry, United Kingdom; 4 Gatsby Computational Neuroscience Unit, University College London, London, United Kingdom; 5 Center for Information and Neural Networks, National Institute for Information and Communications Technology, Osaka, Japan; 6 Computational and Biological Learning Laboratory, Department of Engineering, University of Cambridge, Cambridge, United Kingdom

## Abstract

Humans frequently need to allocate resources across multiple time-steps. Economic theory proposes that subjects do so according to a stable set of intertemporal preferences, but the computational demands of such decisions encourage the use of formally less competent heuristics. Few empirical studies have examined dynamic resource allocation decisions systematically. Here we conducted an experiment involving the dynamic consumption over approximately 15 minutes of a limited budget of relief from moderately painful stimuli. We had previously elicited the participants’ time preferences for the same painful stimuli in one-off choices, allowing us to assess self-consistency. Participants exhibited three characteristic behaviors: saving relief until the end, spreading relief across time, and early spending, of which the last was markedly less prominent. The likelihood that behavior was heuristic rather than normative is suggested by the weak correspondence between one-off and dynamic choices. We show that the consumption choices are consistent with a combination of simple heuristics involving early-spending, spreading or saving of relief until the end, with subjects predominantly exhibiting the last two.

## Author Summary

People often have to trade-off their present wellbeing against their future wellbeing, for example whether to go to an expensive restaurant today or put the money towards a future holiday. Many studies have examined how people make such trade-offs. However, the majority have done so by analyzing choices between one-off future outcomes. By contrast, real-world choices are often made sequentially, with today’s choices influencing the possibilities available tomorrow. This generates decision problems of near limitless complexity. To explore how people approach such decisions in a naturalistic (health-related) setting, we describe participants’ use of a limited budget of relief from moderately painful stimuli over a period of approximately 15 minutes. Participants showed a range of different behaviors, with the majority either conserving relief for the future, or preferring to spread relief evenly over time. Notably no participant consistently consumed the maximum allowable relief at the outset. We show that sequential decision-making behavior cannot easily be predicted from the results of simple one-off choices made at the beginning of the task.

## Introduction

Humans are often required to allocate limited resources across time, for example having to choose whether to go to an expensive restaurant today or put the money towards a future holiday. Economic theory assumes that they do so in a manner which maximizes an intertemporal preference function. This function describes how a decision-maker values events as a function of both their future timing and magnitude [1] and is typically partitioned into two independent sub-functions, an instantaneous utility function, describing the effect of magnitude, and a temporal discount function, describing the effect of delay, with discounted utility of multiple outcomes being summed across time periods [1, 2].

Temporal discount functions are conventionally estimated by eliciting choices between one-off outcomes of varying magnitude at varying delays (the instantaneous utility function is often assumed to take some plausible prior form). It is widely observed that people prefer to receive one-off rewards as soon as possible, consistent with the value of rewards decaying with delay, referred to as positive temporal discounting [for reviews see 3, 4]. However under some circumstances people display an opposite tendency, namely a deferral of reward into the future. In a well-known example, [5] participants were asked to state how much money they would be willing to pay now to receive a kiss from a movie star at varying points in time. The maximum willingness-to-pay occurred when the kiss was scheduled to occur three days in the future, implying a growth in value with delay (over the short term in this example), which is called negative time preference or negative discounting [6, 7]. Negative time preference is also prominent in choices between aversive outcomes, where many people prefer to receive pain (or hypothetical illness) immediately rather than after a delay [5, 8, 9]. An explanation is that the anticipation of future events in itself provides additional present-time utility, termed savoring for positive outcomes and dread for negative ones [5, 10].

According to an assumption of additive discounted utility, an individual’s preferred allocation of rewards over several time periods ought to be predictable from their discount and utility functions derived from choices between the same one-off rewards [2]. In reality the assumption of additive utility is violated. For instance eating a meal reduces the utility of food for some time afterwards. Similar violations occur prospectively too. For example although, as noted, people overwhelmingly prefer sooner one-off rewards to delayed rewards of equivalent magnitude, when the same rewards are framed as sequences people tend to prefer sequences which improve over time—behavior which cannot be reconciled with a single discount function whilst also preserving additive utility [11–15].

The conventional economic model also assumes that humans have the necessary cognitive capacity to optimize their discounted utility. However, when deciding how to allocate reward over several time steps, the number of possible allocation plans grows exponentially as outcomes further into the future are considered, generating decision-problems of considerable complexity [16]. In response to this people apparently adopt simplifying strategies. For instance, transfers into retirement savings plans cluster around the minimum and maximum allowable contributions, as well as around multiples of five dollars, suggesting that investors choose these as convenient ‘rules-of-thumb’ [17]. Such strategies are examples of ‘heuristics’, which are generic, though possibly only partly competent, solutions to classes of problems [18–20]. Notably the use of heuristics can generate behavior that differs from the predictions of conventional economic models of intertemporal choice, in particular leading to on-going choices in a dynamic context that are not consistent with preferences that the decision-maker might exhibit in simpler, e.g. one-shot, contexts [16, 21].

In addition the form of temporal discount function interacts with the ability to execute one’s best-laid plans. A decision-maker with an exponential discount function (and an increasing concave utility function over outcome magnitude) has time-consistent preferences—i.e. will make the same decision between options with different temporal profiles no matter how close or far in time these are [2]. Such a decision-maker would naturally adhere to her plans, however frequently they were re-evaluated. By contrast if the discount function is positive but hyperbolic, as frequently observed [22–24] and/or approximated [25–28] in humans and other animals, then the decision-maker would be expected to exhibit dynamically inconsistent behavior: by seeking immediate reward, they would tend to undo previous long-sighted plans [2, 23, 29, 30], however see [31–33] for an alternative account]. Temporally inconsistent preferences theoretically compound the complexity of planning resource allocations in real-time, since they necessitate a dynamic model of the behavior of future selves [2, 30].

All the difficulties and violations of the conventional economic model of intertemporal choice described above make it unlikely that individuals exhibit fully optimal intertemporal allocations. However very few studies have directly examined resource allocation decisions in real-time, tested the extent to which these are consistent with discount functions derived from one-off choices, or indeed found a parsimonious description that accounts well for actual choices. Thus we designed a task that involved allocating a limited budget in real-time in which we could examine the various forms of inconsistency and explore possible heuristics in a rather open-ended manner. Specifically the task involved choosing how to consume relief from painful stimuli over an extended period of time. In a separate experiment, performed on the same day, [fully described elsewhere [9]], participants made binary choices between different numbers of, and delays to, painful shock stimuli, which were identical to those used for the consumption-savings experiment. We were therefore able to compare the consistency of observed behavior in one-off and dynamic choices.

The existing binary choice study illustrated a range of intertemporal choice behavior; some participants displayed positive discounting, others negative discounting and others discounting very little or not at all. These three patterns would be expected to give rise in the dynamic task to spending relief early, saving relief for the end and spreading relief evenly over time, respectively (the latter assumes a concave utility function for relief). We therefore tested the prediction that if one-off and dynamic choices are consistent, then individuals who positively discounted one-off pains would tend to spend their relief early, those who displayed greater negative discounting (dread) for one-off pains would be more likely to save their relief (to mitigate future punishment), and those who did not discount pain at all would be more likely to spread their relief across time. More specifically we also compared behavior with the optimal predictions of an anticipation-discounting model fitted to the one-off choices. In the light of our findings, we went on to explore more heuristic descriptions of the behaviour that we elicited.

The particular anticipation-discounting functions that we observed can generate non-adherence to past plans of a different form to that entailed by hyperbolic discounting [see 5 and S1 Fig.]. In our case subjects who dread one-off pains are motivated to save more in the present than they would desire to use in the future. Since we did not elicit participants’ plans prior to the experiment, we could not directly test for this. However, to explore the theoretical implications of the model in more detail we simulated optimal consumption choices under various parameterizations of the utility and anticipation-discounting functions, allowing for the possibility that subjects might have different degrees of insight into their future tendencies, being either inaccurate (naïve) or accurate (sophisticated) [34].

We observed prominent tendencies to spread relief across time and to save relief. However higher dread of pain in one-off choices showed no significant correlation with the latter tendency. We found that while some participants displayed behavior consistent with the optimal paths predicted from their dread-discounting functions, several participants exhibited consumption profiles which were not self-consistent. Overall observed consumption behavior was parsimoniously described by *post-hoc* models which assumed that participants combine a set of heuristics to ‘save-now-spend-later’, ‘spread-spending’, and, to a much lesser extent, ‘spend-now-suffer-later’.

## Results

### Relief Consumption Task

The task required participants to perform a series of 60 trials over 14 minutes wherein they were scheduled to receive a number of identical, moderately painful, cutaneous electric shock stimuli on each trial. At the outset, each participant was endowed with a fixed budget of computerized pain relief, an amount insufficient to relieve all shocks in the session. On each trial they were allowed to choose how much relief they wished to use, up to a maximum allowable “dose”. The scenario was embedded within a hypothetical health-related context, and pain relief was described in units of milligrams.

**Figure 1:**
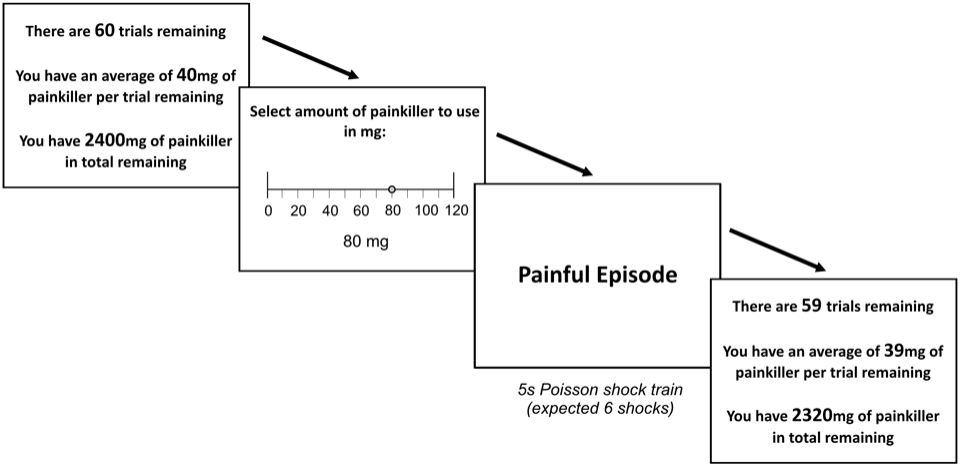
Trial structure of the experiment. Participants entered an experimental run of 60 trials, on which they could expect to receive mildly painful electric shock stimuli on each trial, referred to as painful episodes. By default participants could expect to receive a five second stimulus with 14 brief shocks on each trial, however they were provided with a budget of relief at the outset of the experiment, 2400 “milligrammes” (mg) in total. Each 10mg of relief consumed reduced the expected number of shocks in the stimulus train by one, and was hence insufficient to relieve all the shocks in the session. At the start of each trial a screen indicated the number of remaining trials and the remaining supply of relief. Participants then had the opportunity to indicate how much relief they wished to consume on that trial, up to a maximum of 120mg. The relief was then effective on the immediately subsequent painful episode.

Fig. 1 illustrates the experimental protocol. On each trial, subjects received a number of shocks drawn from a Poisson distribution. Without pain relief, the mean of this distribution was 14 shocks; for every 1mg of relief the subjects spent on a trial, the mean decreased by 0.1 shocks. Subjects were allowed to spend a maximum of 120mg of relief on a trial; this reduced the mean number of shocks to 2, a level termed the ‘baseline pain’. Subjects had to spend within a total budget of 2400mg. Before making their choice, participants were informed of the total relief capital remaining, the number of trials remaining and the mean remaining relief per trial.

In a separate experiment, performed on the same day, [fully described elsewhere 33], participants made binary choices between different numbers of, and delays to, painful shock stimuli (which were identical to those used for the consumption-savings experiment). The unit of time in both experiments was a single trial, of equivalent length in both experiments, and resulted in delays of the order of zero to 15 minutes in both experiments

### Simulating Consumption Behavior

To illustrate the effects of changes to the instantaneous utility and anticipation-discounting functions, we used dynamic programming to simulate optimal behavior on a reduced version of the task lasting 10 time periods (with a budget of 400mg).

**Figure 2:**
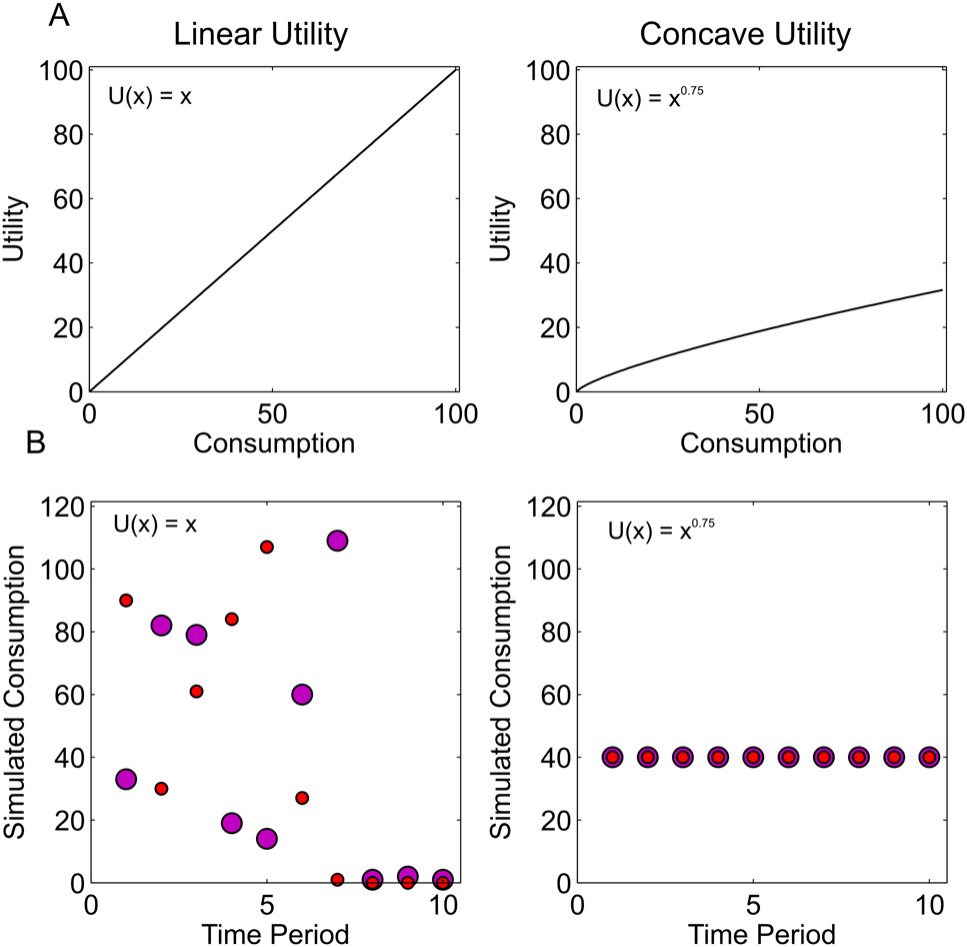
Changes in the instantaneous utility function. **A** Linear and concave utility functions. **B** Simulated optimal consumption paths with no discounting under the two forms of utility function. In each case two sample simulated paths are displayed, to illustrate that, with linear utility there is more than one optimal path. Left panel: under linear utility with no discounting or anticipation all paths which consume the entire budget are equally valued. Consumption is therefore chosen at random from a uniform distribution until the budget is expended. Right panel: concave utility motivates spreading consumption over time.

**Effects of the instantaneous utility function**. Within the standard economic model, the instantaneous utility function can affect the optimal consumption path, even for a decision-maker who treats the same outcome as equally valuable regardless of its timing (Fig. 2).

Let the utility for consuming an amount, *c*, at time, *t*, given current capital, *s_t_* be given by *U*(*c_t_*, *s_t_*) (The only effect of *s_t_* on the instantaneous utility function is to constrain consumption to be less than current capital, such that *c_t_*≤*s_t_*; as a result we abbreviate *U*(*c_t_*, *s_t_*) to simply *U*(*c_t_*), however the above constraint is still implied).

For the special case of linear utility, where *U*(*c_t_*) = *c_t_*, provided there is no discounting or interest rate, all possible consumption paths in which total consumption corresponds to spending the entire capital are equally valued. As a result, at each time step, and each state of capital, all possible consumption levels have equal value. The result is that consumption in the first time period, *c*_1_ could be selected at random from a uniform distribution, in which case, the expected consumption level, *c*_1_, is close to 60 units. Single-period consumption, *c_t_*, then continues in this manner until the capital is entirely consumed (Fig. 2, left panels).

With a concave utility function, here illustrated with $U\left(c_{t}\right)=c_{t}{}^{k},$ where 0 < k < 1, low levels of consumption are relatively more valuable than would be the case under a linear function. Here, the optimal path is to spread consumption evenly across time (Fig. 2, right panels).

**Figure 3:**
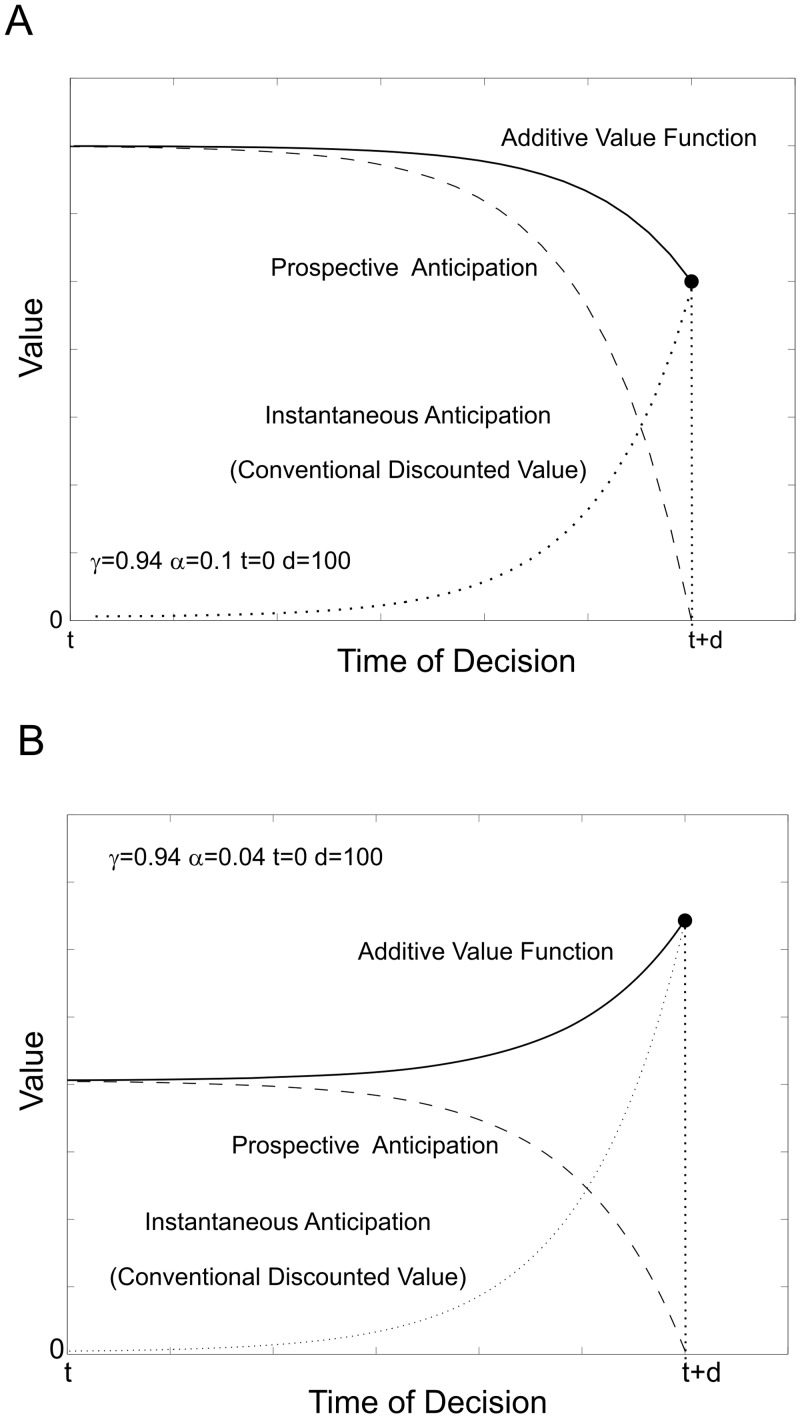
Anticipation-discounting functions. Anticipation-discounting functions are constructed from a linear combination of the conventionally discounted value of an outcome, i.e. its instantaneous anticipation, and the prospective sum of anticipation whilst waiting for the outcome, displayed here for an outcome with positive utility. **A** Where prospective anticipation (savoring) dominates, the overall value of the outcome decreases as it draws nearer, due to decreasing prospective anticipation. **B** Where discounting dominates, the overall value of the outcome increases as it draws nearer due to increasing instantaneous anticipation.

**Anticipation-discounting functions**. In the existing binary choice study, we estimated an anticipation-discounting function, here termed *Δ*(*d*) for each participant, determining how the value of pain depends on its delay, *d*. The anticipation term is computed as the forward-looking sum of exponentially discounted value, with a per-period rate, *γ_c_* (C for consumption) the contribution of which is determined by the parameter *α* (Fig. 3):

$$\Delta \left(d\right)=\gamma_{C}{}^{d}+\alpha \left[\sum_{\tau =0}^{d-1}\gamma_{C}{}^{d-\tau }\gamma_{A}{}^{\tau }\right]$$

The full model, as shown here, allows for the possibility that prospective anticipation is itself discounted by an additional factor, *γ_A_* (A for consumption), representing the extent to which future anticipation is taken into account. Fig. 3 illustrates typical forms for an anticipation-discounting function for a positively-valenced outcome, where *γ_A_* = 1 Where anticipation dominates (Fig. 3A), the overall value is an increasing function of delay; where discounting dominates (Fig. 3B), the overall value is a decreasing function of delay.

**Figure 4:**
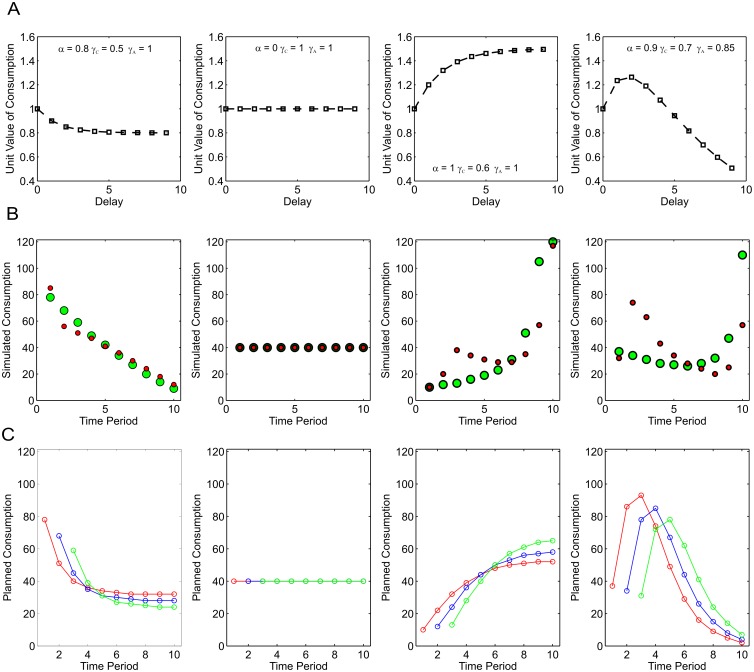
Anticipation-discounting and dynamic utility maximization. **A** Four anticipation-discounting functions. From left to right: predominant discounting, no discounting, predominant savoring, discounted savoring. The parameters of each function are displayed on the plot. **B** Simulated optimal consumption paths under the same four discount functions, with concave utility, *U*(*c*) = *c* ^0.75^ Green circles represent simulated consumption paths for a fully naïve decision-maker (See Main Text). Red circles represent consumption for a fully sophisticated decision-maker. **C** Plans for future consumption made in the first three time periods for a naïve decision-maker. The red circles indicate planned consumption from the perspective of *t* = 1, the blue circles from the perspective of *t* = 2 and the green circles from the perspective of *t* = 3. Where discounting dominates (left panel), the naïve decision-maker consumes more than planned, where savoring dominates (right hand two panels), the naïve decision-maker consumes less than planned.

Fig. 4 plots predicted consumption paths under four possible parameterizations of the anticipation-discounting function (Fig. 4A), under both full naivety or full sophistication, for a concave utility function: $U\left(c_{t}\right)=c_{t}{}^{0.75}$. Complete sophistication entails that the agent at *t* = 1 knows that a future decision-maker, for example at *t* = 11 will apply the same degree of discounting to periods *t* = 11,12,13… and so on as the agent currently applies to periods *t* = 1,2,3… and so on. Naivety by contrast would entail that the agent at *t* = 1 assumes that the decision-maker at *t* = 11 will apply the same discount factors to periods *t* = 11,12,13… and so on as the agent currently applies to those time periods. Given dynamic inconsistency in the discounting function, a naïve agent would be expected to change their plans at each time step.

It can be appreciated from Fig. 4B that, where discounting dominates (first column), optimal consumption is decreasing. With no discounting (second column), optimal consumption is even over time, owing to concave utility. Where anticipation dominates (third column), the predicted consumption path is increasing. Where anticipation is itself discounted (*γ_A < 1;_* fourth column) non-monotonic consumption profiles result.

Where there is a degree of savoring (*α*>0), the consumption paths for naïve and sophisticated consumers diverge, albeit subtly in some cases. The underlying dynamic inconsistency is illustrated in Fig. 4C, which plots consumption plans made at the first three time periods for fully naïve agents. Rather than consumption itself, these plots depict the naive plans for future consumption from the current time-period onwards. Where discounting dominates (left column), inconsistency similar to that implied by hyperbolic discounting results: consumption at the next period turns out to be greater than planned. Where savoring dominates (right hand two panels), the naïve decision-maker consumes less than planned. A sophisticated agent takes these future discrepancies into account and adjusts their plan accordingly.

To summarize, normative considerations justify at least three obvious qualitative classes of relief spending—increasing, decreasing and flat (or spreading). They also make strong predictions about the relationship between single- and multi-period decisions, and potentially the effect of degrees of game-theoretic sophistication. Our experiment was designed to test for these classes, but, motivated by the complexity of planning, also to provide insights into possible heuristics.

### Observed Consumption of Relief

**Figure 5:**
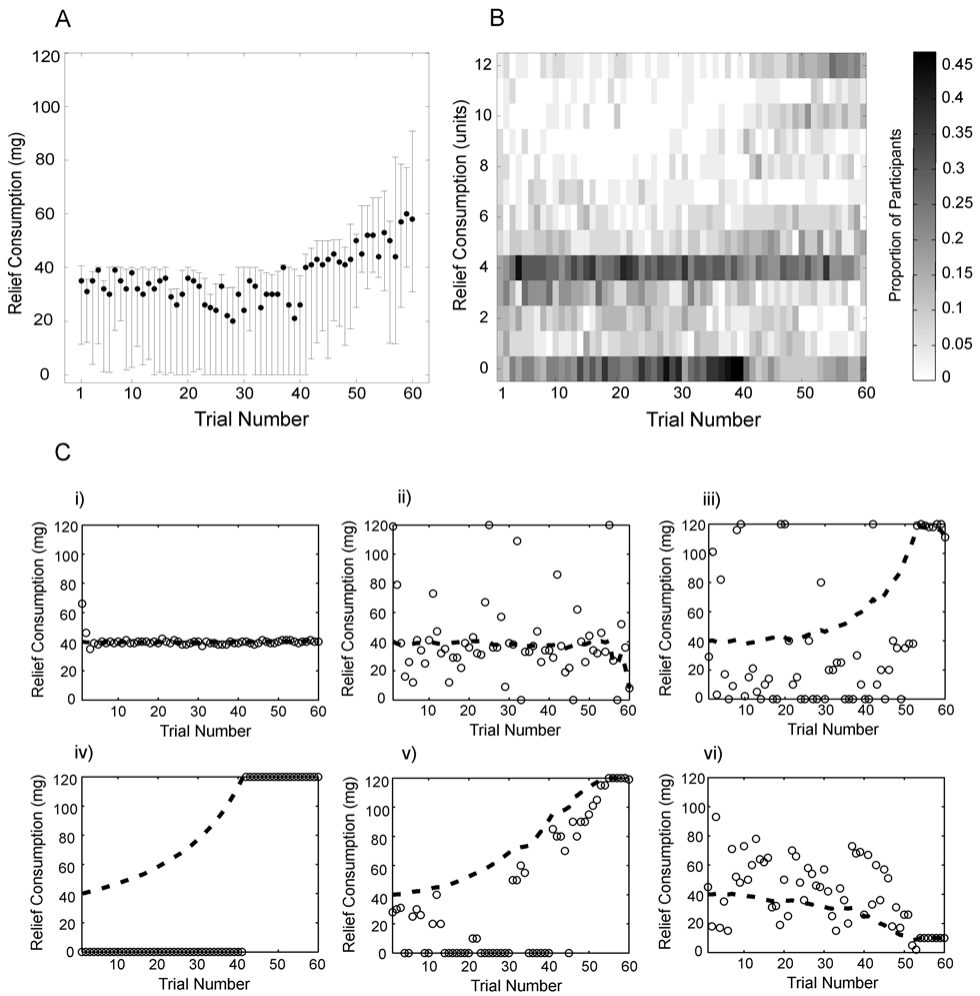
Observed distribution of relief consumption across time. **A** Median relief consumption on each trial at the group level is indicated by the solid black circles. Error bars indicate the upper and lower quartiles of consumption. **B** The distribution of group level consumption. The intensity of the grey bars represents the proportion of the 30 participants included in the analysis choosing to consume each amount of relief on a particular trial. Relief is expressed in units, produced by rounding the raw consumption choices to the nearest 10 units of relief. A tendency to conserve relief is evident from below-average consumption over the first 40 trials and above-average consumption over the last 20 trials. A tendency to spread relief across time is evident from the high proportion of choices to spend 4 units of relief, the mean rounded relief per trial over the whole experimental run. **C** Consumption paths from six sample participants. Hollow circles denote the consumption choices on each trial. The bold dashed line denotes the mean relief remaining at the start of each trial. These six participants are selected as representative of the key patterns observed. The first two participants (i and ii) on spread relief over time. The subsequent three participants (iii-v) predominantly conserve relief, as evidenced by an increase in the mean relief remaining per trial over time. Participant vi) consumes above the mean relief remaining towards the start of the session and subsequently adjusts consumption downward.

We tested 35 participants, of whom 5 had to be excluded from analysis (see Methods for details). The experimental data (S1 Dataset) consisted of the number of units of relief consumed on each trial by each participant. Fig. 5A plots the median consumption of relief on each trial at the group level (*N* = 30, bars indicate the interquartile ranges). Across subjects, the profile of consumption is increasing over time, showing the tendency for relief to be saved for towards the end of the experimental session. Robust linear regression on all choices made by all subjects (*N* = 1980), using iteratively reweighted least squares with a bi-square weighting function, demonstrated a significantly positive effect of time on relief consumption (*β* = 0.47, *p*<0.001).

However, the group-level presentation of the data conceals the complexity of subject-specific choices. To examine this we calculated the proportion of participants choosing a particular level of consumption on each trial. To reduce the computational complexity of the subsequent modeling analysis (necessary when fitting more complex models using dynamic programming), relief consumption was rounded to the nearest 10mg, creating 13 possible spending choices on each trial (0 to 12). We refer to each rounded centigram simply as a ‘unit’ of relief. The observed distribution of rounded relief-consumption at the group level is displayed in Fig. 5B. Darker bars indicate a higher proportion of participants choosing a given consumption level on each trial. There were very few choices to consume close to the maximum quota of relief early in the experimental session. Rather, higher intensities corresponding to spending close to zero relief in the first 40 trials, and above-average consumption across the final 20 trials, demonstrated that participants tended to conserve relief for the final portion of the session, which would be consistent with savoring. Since there was a budget of 240 rounded units of relief, to be allocated across 60 trials, even spreading of relief would entail spending 4 units per trial. Notably, high intensities corresponding to spending close to 4 units of relief indicate that participants also demonstrated a tendency to spread relief across time, which would be consistent with participants having concave utility for relief. There is also a weak tendency to sample the maximum allowable quota of relief throughout the experimental run. An additional interesting feature is that participants were more likely to consume close to the mean relief remaining early in the experiment, tending to switch to consuming zero relief during the middle of the experiment.

S2A Fig. plots raw consumption choices (in mg) as a series of histograms over time, illustrating that multiples of 10mg are over-represented. This suggests that participants used strategies to reduce the dimensionality of the task, rather than performing optimization at the native resolution. When rounded consumption (in units) is also plotted in this manner (S2B Fig.), choices to consume zero relief or 4 units of relief are prominent. Raw data for the 30 participants included in the analysis are displayed in S3, S4 and S5 Figs. At the individual level, participants appeared to display one or more of the above three tendencies, though strikingly, no participant systematically consumed close to the maximum available relief at the outset of the experiment. To illustrate this, consumption profiles from six sample participants are displayed in Fig. 5C, overlaid with the mean relief remaining per trial (dashed lines), termed *ρ_t_*. This quantity (displayed to participants on-screen before each choice) is given by the total remaining relief on that trial, *s_t_*, divided by the number of trials remaining:

$$\rho_{t}=s_{t}/\left[60–\left(t-1\right)\right]$$ 2)

For any trial during the experiment, consuming exactly *ρ_t_* units of relief on every remaining trial would entail even consumption of relief over the remainder of the experiment.

### Predicting Consumption from One-Off Choices between Delayed Pains

Although the subjects exhibited the same qualitative patterns of behavior as expected from the normative accounts (Fig. 4), this does not mean that each subject’s own choices were consistent with their own one-off preferences. To compare one-off and dynamic behavior, we first derived summary measures of behavior on both tasks. In the one-off choice task, the frequency of choosing sooner pain indicates the extent of negative time preference, and is a correlate of dread. As described previously, [9], one-off choices between delayed pains were elicited under two descriptive frames, a ‘pain’ frame, in which outcomes were described as an increase in the expected number of shocks above the baseline level of pain, and a ‘relief’ frame, in which the same outcomes were described as a decrease in the expected number of shocks from a maximum level of pain. The latter description corresponds to that used in the relief consumption experiment. Nevertheless we examined the relationships between dynamic relief consumption behavior and sooner choice frequency on both frames. The signed slope of the dynamic consumption path (fitted with least-squares linear regression), is a measure of the overall tendency to conserve relief, while the absolute magnitude of the slope is a measure of the deviation, in either direction, from even spreading of relief.

**Figure 6:**
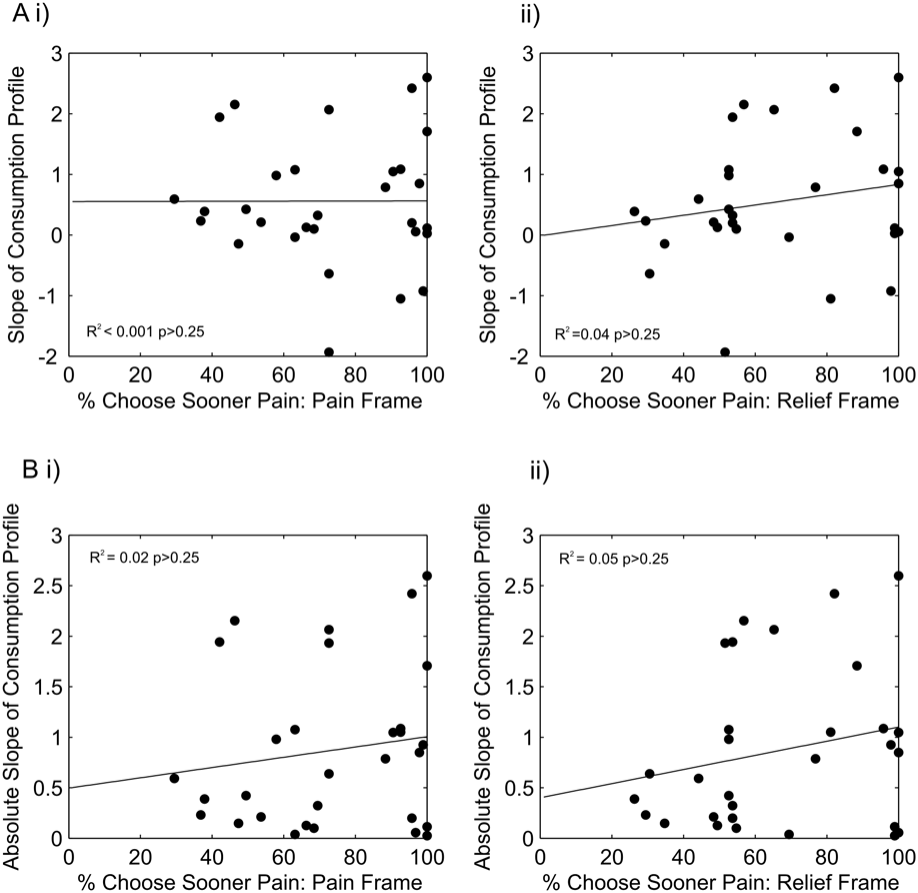
Relationships between one-off (binary) and dynamic intertemporal choices. The frequency of choosing sooner pain in the binary intertemporal choice experiment which provides a summary behavioral measure of dread, in both pain **(i)** and relief **(ii)** frames (see main text), is plotted against: **A** the slope of the spending profile in the dynamic consumption task (positive slope indicates saving relief) and **B** the absolute slope of the spending profile (a measure of deviation from even spreading of relief). Solid lines indicate a linear least-squares fit through the data. There are no significant relationships between the behavioral metrics on the two tasks.

Contrary to a normative account, we observed no significant positive relationship between the tendency to dread (on either frame) and the slope of the consumption path (Fig. 6A; *p*>0.25, *N* = 30), although there was a trend in this direction for the relief frame choices (Pearson *r* = 0.2). Neither was there a significant relationship between dread and the tendency to spread relief over time (Fig. 6B; *p*>0.25, *N* = 30).

**Figure 7:**
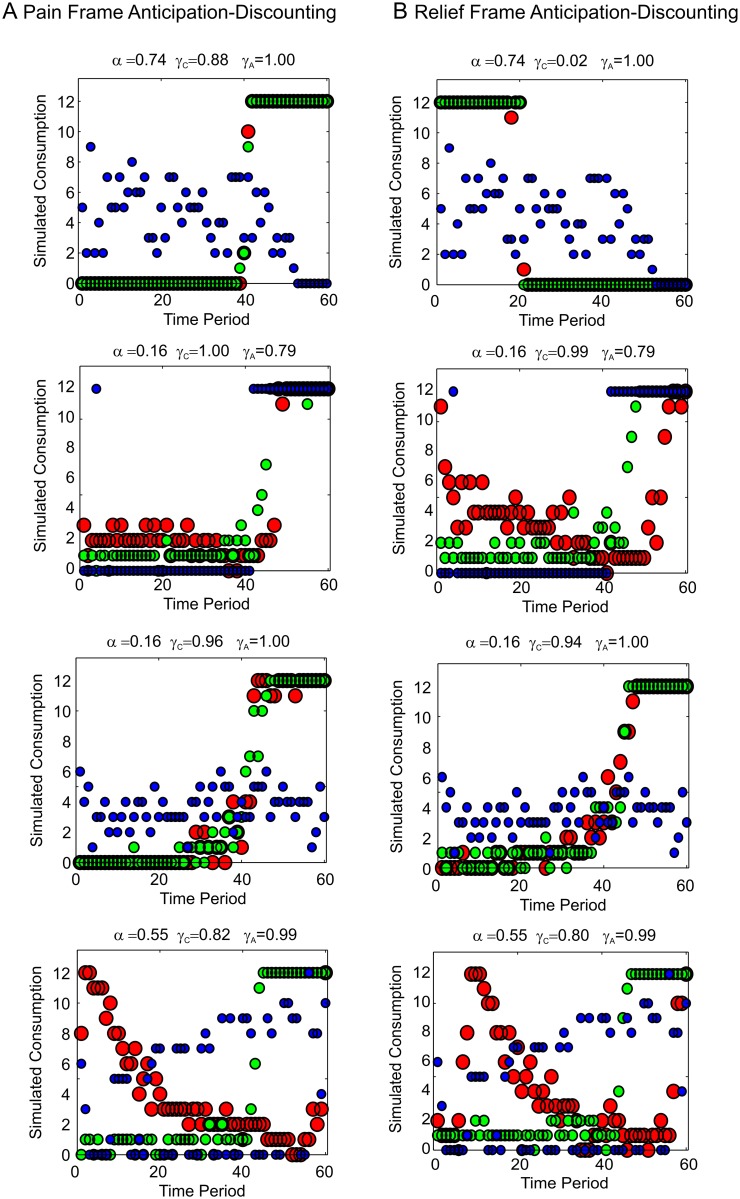
Optimal consumption paths predicted from anticipation-discounting functions derived from binary choices. Naïve (green circles) and sophisticated optimal (red circles) paths, derived from binary intertemporal choices in both pain (left column, A) and relief (right column, B) frames with softmax *β* = 10 and *U*(*c*) = *c* ^0.75^ are overlaid on observed consumption paths (blue circles) for 4 sample participants.

For those participants for whom estimates of anticipation-discounting functions were available from one-off choices (*N* = 23; see Methods), we compared observed relief consumption with the predicted consumption profiles for both naïve and fully sophisticated agents with this anticipation-discounting function, assuming a concave instantaneous utility function for relief, *U*(*c*) = *c* ^0.75^. We considered the policy to be a softmax function of the underlying values, setting the inverse temperature parameter to an arbitrary value for all participants (*β* = 10), whilst fixing the anticipation-discounting parameters to those previously derived from one-off choices. Sample results for four participants are plotted in Fig. 7. It can readily be seen that the observed consumption profiles (blue circles) in some instances diverge markedly from the predictions (sophisticated predictions, red circles; naïve predictions, green circles).

**Figure 8:**
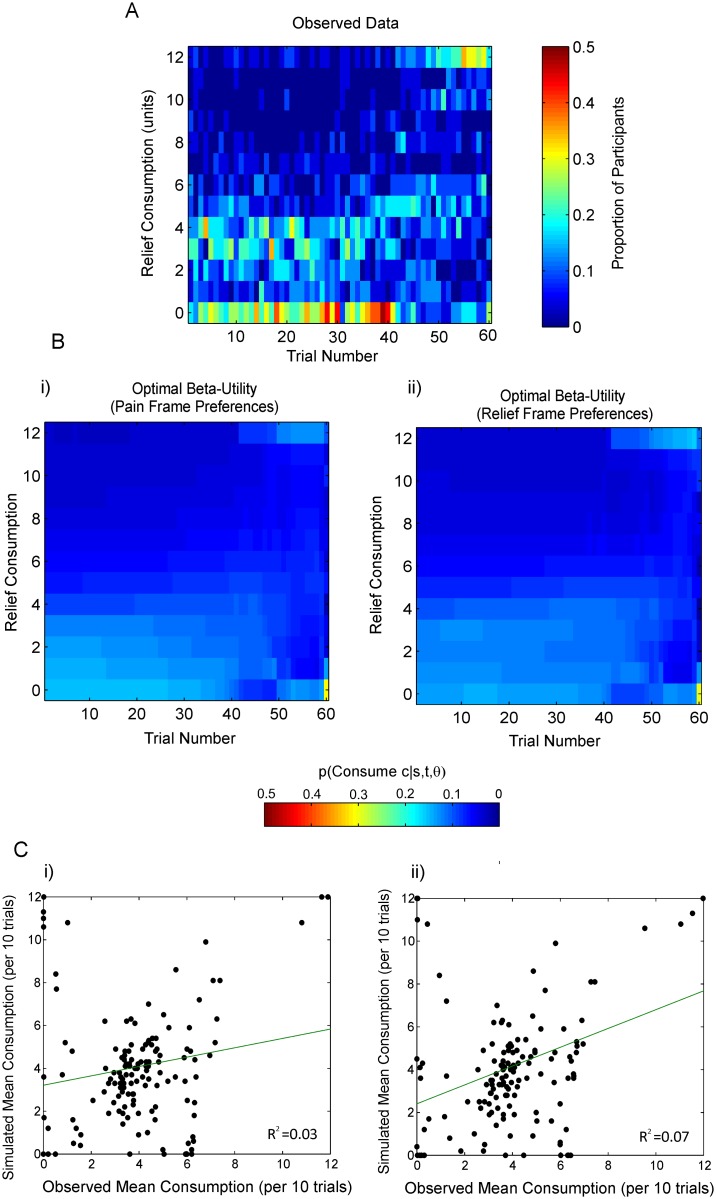
Fits of the anticipation-discounting model with variable utility and choice randomness. **A** The observed distribution of consumption at the group level by participants for whom anticipation-discounting functions derived one-off choice tasks were available (*N* = 23). Warmer colors indicate that a higher proportion of participants chose to consume that amount of relief on a particular trial. **B** Group-Level distribution of relief consumption predicted by the optimal model and modifications to it. These plots denote the mean probability across all participants of consuming an amount of relief, *c_t_*, on each trial, *t*, given a vector of the total remaining relief for each participant on each trial, *s_t_*, *s_t_*_+1_, *s_t_*_+2_, … s_T_, at the maximum likelihood parameters, *θ*, of each model. i) Anticipation-discounting functions derived from one-off pain frame choices, with the softmax temperature, beta, and utility parameters freely fitted. ii) Anticipation-discounting functions derived from one-off relief frame choices, with the softmax temperature, beta, and utility parameters freely fitted. **C** The proportion of variance explained by each model. Mean predicted consumption levels simulated from the maximum likelihood parameterizations of each model over each 10 trials of the experiment for each participant are plotted against the same metric derived from the observed data.

It is possible that variability in the utility function and softmax temperature parameters could account for some of the observed differences between dynamic and one-off choice settings, whilst preserving the basic intertemporal preferences. To explore this we implemented a model in which the softmax inverse temperature, *β*, and the exponent governing the utility function, *k*, were fitted freely, whilst holding the previously-derived anticipation-discounting parameters constant. To fit the model we used constrained non-linear optimization to find subject-specific parameters, which maximized the log-likelihood of the observed consumption paths for each participant, given their remaining capital on each trial. The observed group level distribution of consumption in the same 23 participants is displayed in Fig. 8A, for comparison with the distribution predicted by the model. The latter, formed by taking the mean across the likelihood distributions for individual participants, is shown in Fig. 8Bi (pain frame preferences) and 8Bii (relief frame preferences). Although the optimal preferences predict saving of relief at the group level, they underestimate the tendency to spread relief over time, even allowing for concave utility, and the fitted policies are relatively imprecise. To estimate the proportion of variance in the observed data accounted for by the models, we found the mean consumption level for each participant across each 10 trials of the experiment, before calculating the same measure by simulating 10000 consumption paths resulting from the maximum likelihood parameterization of the model. As shown in Figs. 8Ci (pain frame preferences) and 8Cii (relief frame preferences), there was a significant positive relationship between predicted and observed consumption paths (robust regression, pain frame: *β*_1_ = 0.22, *p*<0.001; relief frame: *β*_1_ = 0.44, *p*<0.001). However least squares fits indicated that the model accounted for only a relatively small proportion of the observed variance (pain frame *R* ^2^ = 0.03, relief frame *R* ^2^ = 0.07).

### Modeling Relief Consumption Using Heuristics

Given that consumption behavior showed only weak correspondence with the predictions of anticipation-discounting as derived from one-off choices, we tested alternative generative accounts. This analysis was performed *post hoc*, and we focused on characterizing simple computations that might feasibly have produced the observed consumption choices. To do so we assumed that participants implemented the three main behavioral tendencies, namely spending, spreading and saving relief, as heuristics.

The first model, which we termed the Direct Action Heuristic model, proposed that participants implemented the three observed behavioral tendencies directly, with choices between them governed by propensities. The three are termed spend-now-suffer-later (with propensity *M*_spend_), spread-spending (with propensity *M*_spread_), and save-now-spend-later (with propensity *M*_save_). The extent to which observed relief consumption, *c_t_*, fell below the mean relief remaining on each trial, *ρ_t_*, is given by:

$$d_{t}=\rho_{t}-c_{t}$$ 3)

Positive *d_t_* entails using less than the mean relief remaining per trial, while |*d_t_*| indicates the extent of deviation from spreading. Formally, the three heuristics were defined as (see Methods for details):

$$M_{\text{spend}}\left(s_{t},c_{t},t\right)=c_{t}$$ 4)

$$M_{\text{spread}}\left(s_{t},c_{t},t\right)=-\left|d_{t}\right|$$ 5)

$$M_{\text{save}}\left(s_{t},c_{t},t\right)=\{\begin{array}{cc} d_{t} & if\;\rho_{t}<12\\ 0 & otherwise \end{array}$$ 6)

*M*_spend_ formalizes a spend-now-suffer-later heuristic, by assuming linear utility for relief consumption, and thus a propensity to consume the maximum allowable relief. *M*_spread_ formalizes a spread-spending heuristic, by penalizing deviations from the mean relief remaining, and so generates a propensity to spread relief over time. *M*_save_ formalizes a save-now-spend-later heuristic, by assigning higher value to consuming less relief, provided that the mean remaining relief per trial is less than the maximum possible consumption level. *M*_save_ therefore generates a propensity to consume as little relief as possible until there is sufficient remaining relief to reduce pain to the baseline level for the remainder of the experiment, at which point the remaining heuristics encourage spending this quantity.

The three action propensities were implemented as separate policies, each with a unique softmax inverse temperature parameter; the final probability of consuming each level of relief was assumed to arise from a weighted average across these policies with weight for a policy determined by its inverse variance (see Methods). As previously, to fit the model we used constrained non-linear optimization to find subject-specific parameters, which maximized the log-likelihood of the observed consumption paths for each participant (*N* = 30), given their remaining capital on each trial.

**Figure 9:**
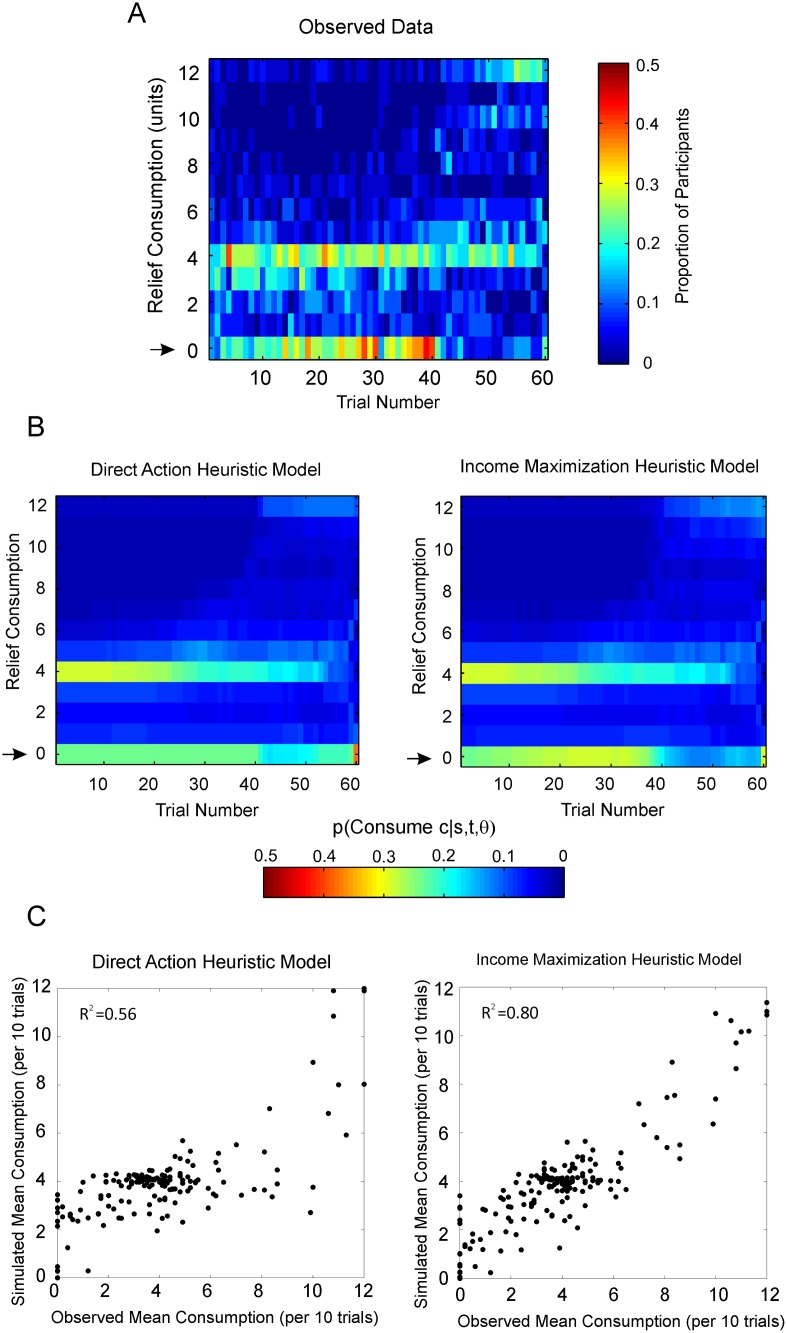
Heuristic model fits. **A** The observed distribution of consumption by all 30 participants included in the analysis. Warmer colors indicate that a higher proportion of participants chose to consume that amount of relief on a particular trial. Black arrows indicate spending zero relief, which becomes more prominent during the middle of the experiment. **B** Group-Level distribution of relief consumption predicted by alternative heuristic models. These plots denote the mean probability across all participants of consuming an amount of relief, *c_t_*, on each trial, *t*, given a vector of the total remaining relief for each participant on each trial, trial, *s_t_*, *s_t_*_+1_, *s_t_*_+2_, … s_*T*_, at the maximum likelihood parameterization, *θ*, of each model. The Direct Action model combines the three key observed behavioral tendencies as heuristics to either spend close zero relief until the mean relief remaining reaches the maximum allowable spend (save-now-spend-later), to spending close to the mean relief remaining per trial (spread-spending) or close to the maximum allowable relief (spend-now-suffer-later). The Income Maximization model extends this model, such that the saving tendency is implemented as the attempt to dynamically maximize the mean remaining relief per trial, over a limited future horizon. This model captures the relatively greater tendency to save relief during the middle of the experiment (as indicated by the black arrows). **C** The proportion of variance explained by each model. Mean predicted consumption levels simulated from the maximum likelihood parameterizations of each model over each 10 trials of the experiment for each participant are plotted against the same metric derived from the observed data. Least squares fits indicate an R-squared value of 0.56 for the Direct Action model and 0.80 for the Income Maximization model.

The group-level distribution of observed consumption choices is reproduced in Fig. 9A, for comparison with the model fits. The distribution predicted by the Direct Action heuristic model is displayed in the left-hand panel of Fig. 9B. The model provided a parsimonious summary of observed consumption choices, albeit not convincingly capturing the observation that some participants were more likely to consume close to the mean relief remaining per trial (*ρ_t_*)near the start of the experimental run, before switching to conserve relief.

The above pattern might have several different explanations. One simplification in the model is that the explicit relative weightings of the heuristics are assumed to be constant. However, participants may have adopted the spread-spending heuristic at the outset, before learning the extent that they were able to tolerate pain as the experiment progressed then switching to save-now-spend-later (see S1 Text). Similarly they may have consumed the mean relief at the outset as a default option, until they learned to trust the experimental setup. A further possibility is that participants, rather than using a save-now-spend-later heuristic directly as defined above, may have sought to maximize the mean relief remaining per trial (*ρ_t_*) over the near future: since saving relief would have more immediate effect on *ρ_t_* later in the experiment compared with at the start, the propensity to save would be expected to increase as the experiment continued.

The data do not admit a direct distinction between the above hypotheses. However in order to illustrate one of the possibilities we fit a modified version of the above model in which the save-now-spend-later heuristic described above is replaced with a heuristic to maximize *ρ_t_* over a limited future horizon, which we term an income maximization heuristic (and eponymous model). Thus *M_save_* in this model was replaced by an action-value function, which described the value of consuming an amount, *c_t_*, at the current capital level, *s_t_*, and time period, *t*, given knowledge of the future policy for action, *π* (see Methods). In other words this model assumed that participants were in part attempting to maximize the expected mean relief remaining per trial, akin to maximizing their expected income. To account for limited computational resources, we incorporate a probability, 1–*γ*, that the decision-maker terminates their search at every level deeper into the tree (the *γ* parameter is mathematically equivalent to an exponential discount rate). We fitted this part of the model using dynamic programming. The remaining two action propensities, *M*_spend_ and *M*_spread_ were implemented in the same manner as the Direct Action model, and policies were combined using the same weighting method.

The distribution of consumption at the group level predicted by the Income Maximization model is shown in the right-hand panel of Fig. 9B. It can be seen that this model accounts for the tendency to save relief being higher during the middle part of the experiment. As expected, the Income Maximization model produced an improvement in Bayesian Information Criterion (*BIC*) [63, see Methods], of 78 at the group level over the Direct Action model. The *BIC* favors models with higher likelihood estimates and penalizes increasing model complexity, where lower values of *BIC* indicate a more favorable model fit. (Notably the Income Maximization model was optimized *post hoc* to account for a particular feature of the observed data, and therefore our primary goal was not to compare the two heuristic models). The maximum-likelihood model fits of the Income Maximization model for the six participants whose data is displayed in Fig. 5 are shown in S7 Fig.

The proportion of variance explained by the models at the ten-trial resolution is shown in Fig. 9C. Least squares fits indicate *R* ^2^ = 0.56 for the Direct Action heuristic model and *R* ^2^ = 0.80 for the Income Maximization heuristic model.

**Figure 10:**
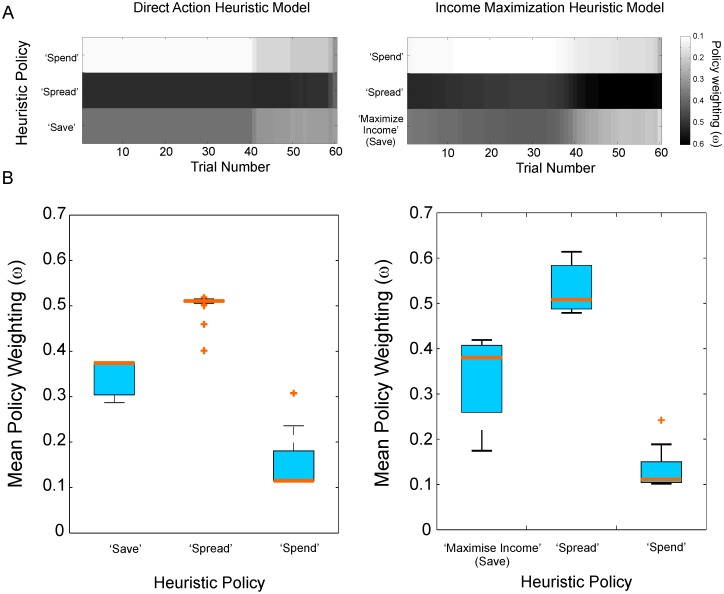
Policy weightings of heuristic models. **A** Mean weighting across subjects on each of the three policies (see Methods) on each trial under the maximum likelihood fits of the Direct Action (left) and Income Maximization (right) models. Spend-now-suffer-later has low weighting early in the experiment. Spread-spending has high weightings throughout. For the income maximization model, saving is weighted most highly in the middle part of the experiment. **B** Box plot showing distribution over participants of policy weightings, averaged over all trials of the experiment. Saving and Spread-Spending heuristics dominate.

To illustrate the contribution of each of the three heuristics, the policy weightings of the two heuristic models are displayed in Fig. 10. The Income Maximization model results in a larger relative weight being placed on saving during the middle half of the experiment (Fig. 10A). Also throughout the experiment saving (save-now-spend-later and income maximization) and spread-spending receive considerably higher weightings (Figs. 10A and 10B) than spend-now-suffer-later. The maximum likelihood parameters for the Income Maximization model are listed in S1 Table.

Finally we implemented a model in which participants could combine optimal choices according to anticipation-discounting function with the above heuristics, attributing deviations from optimality to the use of heuristics. Here, the heuristics can be viewed as attractions towards spending salient quantities of relief, and/or embodying additional valuation processes which play a role in the dynamic task over-and-above anticipatory utility, such as adaptation. In this model participants (*N* = 23) were assumed to perform dynamic utility maximization, according to their previously-derived anticipation-discounting functions, whilst also being biased towards spending either zero, the mean remaining or the maximum relief. Biases were implemented by augmenting the values of consuming these quantities (with Gaussian blur either side, see Methods). The extent of each bias was governed by a weighting parameter, giving rise to three parameters ω_*min*_, ω_*mean*_ and ω_*max*_. The softmax inverse temperature, *β*, and the exponent governing the utility function, *k*, were also freely fitted. We used intertemporal preferences from the relief frame here, since these showed closer correspondence with the observed data. Our aim here was to illustrate formally that deviations from optimality can be parsimoniously described by postulating the use of heuristics. The results are displayed in S7 Fig., showing that the model captures a substantial proportion of the observed variance (*R* ^2^ = 0.83). This model produced an improvement in *BIC* of 430 over the Income Maximization heuristic model, for the subset of 23 participants for whom anticipation-discounting functions were available, suggesting that the addition of utility optimization improved the fit quality over heuristics alone. However both the set of intertemporal preferences and the heuristics for this model were chosen *post hoc*, making it potentially susceptible to over-fitting.

## Discussion

Decision-makers routinely plan the allocation of limited resources over time. Economic theory proposes that they should do so in a self-consistent manner [1]. That is, allocation choices made sequentially ought to be predictable from choices between equivalent one-off delayed outcomes. We tested this by observing the real-time consumption of a limited budget relief from a series of 60 painful stimuli in the laboratory, over the course of approximately 15 minutes, in a group of participants whose intertemporal preferences for one-off future pains of the same nature had been elicited previously. We also sought to provide parsimonious descriptions of the observed behavior in this complex dynamic task.

Tendencies to consume the minimum allowable relief early on, thus saving for the end, and to consume close to the mean remaining relief were prominent, with several participants alternating between these two tendencies. Consistent with retirement-savings decisions, [17] choices to spend multiples of 10mg of relief were over-represented in the data. No participant systematically consumed close to the maximum available relief at the outset of the experiment, as conventional temporal discounting would predict. Whilst two out of the thirty participants analyzed did generate declining profiles of relief, these two participants also showed trial-to-trial variability in consumption, suggesting that they may have chosen consumption levels largely at random (with the decline resulting from exhausting the budget).

We observed no significant correlation between a preference for sooner pain in one-off choices and the tendency to save relief in the dynamic task, although there was a trend towards a positive relationship. When anticipation-discounting functions derived from one-off choices were used to generate optimal consumption paths, whilst freely fitting the utility function and the degree of choice randomness, there was a weak but statistically significant positive relationship between the observed and predicted paths. We conceptualized deviations from optimality in terms of heuristics, rule-of-thumb strategies designed to ease computational demands. We generated putative heuristics *post hoc*, in light of the three observed behavioral tendencies, finding that consumption behavior was well-described by a combination of three corresponding simple rules, namely save-now-spend-later, spread-spending and spend-now-suffer-later, implemented as direct action propensities. However this Direct Action Heuristic model failed to capture an interesting dynamical feature of the data, namely the tendency of several participants to commence saving relief during the middle of the experiment. A possible explanation for this phenomenon posits that rather than directly implementing a save-now-spend-later heuristic, participants attempted to maximize their mean remaining relief (income) over the near future. This Income Maximization Heuristic model outperformed its Direct Action counterpart and accounted for a substantial proportion of the observed variance. Finally we showed that superimposing the heuristics on dynamic utility maximization improved model fits over the heuristic models alone.

At an empirical level the three heuristics serve as parsimonious descriptions of the observed behavior. At a computational level we envision the heuristics as resulting from attractions towards spending salient quantities of relief, hence their usefulness as simplifying strategies, but also as approximating, through their dynamics, more fundamental valuation processes. It is important to note here that the three heuristics can generate behavior indistinguishable from what is optimal under several possible utility functions. For this reason, based on the current data we cannot draw firm conclusions regarding the fundamental valuation processes; however, we outline below a broad framework for categorizing the possible underlying psychological phenomena in terms of relative (reference-dependent) and absolute valuation processes (Table 1).

**Table 1:** Putative mechanisms underlying *save-now-spend-later, spend-now-suffer-later* and *spread-spending* heuristics.

	**Valuation Mechanism**	
	***Relative***	***Absolute***
**Save-Now-Spend-Later**	Adaptation	Anticipation
		Risk aversion
**Spend-Now-Suffer-Later**	Sensitization	Temporal Discounting
**Spread-Spending**	Loss aversion	Risk aversion (concave utility)

The psychological processes motivating the choice of heuristics might be classified as both relative (reference-dependent) and absolute valuation mechanisms. Relative valuation mechanisms include adaptation to current consumption levels, sensitization to repeated punishment and loss aversion. Absolute valuation processes include anticipatory utility, temporal discounting and risk aversion.

Relative valuation processes involve comparison of outcomes against an assumed baseline, or reference-point [35, 36]. Relative valuation might generate a preference for improvement over time, if consumption levels are compared with those that precede them, leading people to choose deliberate privation in order to increase the hedonic impact of subsequent consumption [10, 13, 37]. This would be consistent with existing findings showing that, due to psychological adaptation to the current pain level, a moderate intensity pain can appear more severe when following a low intensity pain than when following a high intensity pain [38]. The opposite effect may also occur, namely sensitization to repeated high level pain, leading participants to occasionally consume the maximum relief as ‘respite’. A further possibility is that decreases in consumption from one time period to the next are valued as more negative than equivalent increases are valued positively, i.e. loss aversion [39–41]. Loss aversion would be expected to further penalize deviations from either even spreading or saving, for the reason that any increases in consumption above even spreading inevitably lead to future decreases [14]. Notably loss aversion itself may represent the heuristic assumption that losses predict further decline, which if unchecked carries the risk of eventual ruin.

Absolute valuation processes might also explain spreading and saving of relief. Firstly a preference for spreading rewards or punishments evenly could arise out of a desire to avoid being left with little or no reward, or high levels of punishment, in some time periods. As demonstrated here through simulation, this desire can be formalized as a non-linear utility function for both reward and punishment, i.e. decreasing marginal (concave) utility for reward and increasing marginal (convex) disutility for punishment. [for a description of how a non-linear instantaneous utility function can affect inferred discounting see 42]. Secondly, saving behavior might result from either anticipatory utility [5, 9, 10], or uncertainty regarding future resources [43]. An interesting direction for future work will be to attempt to prime these mechanisms individually within a more constrained task.

The plurality of possible mechanisms contributing to dynamic behavior might in part explain the low correlation between the anticipatory utility of pain in one-off choices and the tendency to save relief [13]; in particular, relative valuation processes might be expected to play a greater role in the dynamic task, where transitions between outcomes are more salient. Notably, this kind of context-dependent engagement of valuation mechanisms lies outside the conventional economic model of intertemporal preferences, in which the effect of delay is encapsulated by a unitary discount function (if the parameters of the discount function are entirely context-dependent, the model ceases to make useful predictions).

Since we developed the heuristic models after observing the data, they require independent validation in related experimental contexts to establish their generalizability. For example, presenting participants with on-screen details of mean relief remaining may have primed a *spread-spending* heuristic out of a desire to conform to the demands of the experiment. However, in support of the heuristic models proposed here, existing studies show that similar heuristics appear evident in other settings. The widespread use of such strategies suggests common underlying valuation processes. In particular, preferences for spreading rewards evenly across time and for improvement over time are evident in choices between predetermined sequences of outcomes, including wages [15], health [11, 12] and other desirable or undesirable events such as dining at a favorite restaurant or scheduling a visit from a troublesome relative [14]. Loewenstein and Prelec [14] propose a model for classifying these preferences, which resembles the Direct Action heuristic model used here, albeit not in the context of whole sequences of choices over time, as here.

An interesting direction for future work is to determine how choices made in advance between pre-determined sequences differ from choices made sequentially. If people have time-inconsistent preferences, choosing in advance may offer an opportunity for pre-commitment [44–46]. For example, Read and colleagues provide evidence that sequential choice promotes the selection of options that yield small immediate rewards (‘vices’), while choosing the sequence in advance encourages the selection of long-term rewarding options (‘virtues’), a pattern consistent with hyperbolic discounting [47]. As demonstrated here (as well as in existing studies), the anticipation-discounting functions described previously for one-off choices predict a novel form of inconsistent choice, distinct from that of hyperbolic discounting, which entails the perpetual deferral of consumption (S1 Fig.) [5]. It is unclear whether such behavior is manifest in real-time, or indeed influences the kind of consumption choices demonstrated here.

Finally, an advantage of this study is its face validity as a naturalistic scenario. The prominent tendencies to either save relief or to spread relief across time here may have implications for dynamic health-related decision-making in the field. In the UK, personal budgets for healthcare have recently been piloted, potentially giving an individual control over a component of their health spending [48]. Our results suggest that individuals differ considerably in their preferred budget allocations over time. From a policy perspective, such individual differences will be interesting to examine as more data on the use of personal health budgets emerge [49]. Applied measures of choice over time have tended to focus exclusively on one-off choice paradigms [50–53], and the modelling of dynamic decision-making tasks suggests a novel and quantitatively rich behavioral predictor.

In summary we examined how people allocate resources for mitigation of punishment, showing that behavior is not clearly consistent with conventional economic models of intertemporal preference, but is consistent with a simple set of heuristics that encapsulates saving in the present to spend in the future, spreading consumption out evenly over time and (less prominently) spending in the present at the expense of the future. We note that similar behavior is seen in choices between predetermined outcome sequences.

## Methods

### Ethics Statement

The research received approval from the National Health Service National Research Ethics Service, Central London Research Ethics Committee 3 (Ethics number 08/H0716/6, Amendment AM1). All participants gave informed consent before taking part in the study.

### Relief Consumption Experiment

**Participants**. Thirty-five participants (18 females) took part in the study, with full informed consent. Participants were recruited via an advertisement on the website of the University College London Psychology Subject Pool. The experiments were carried out at the Wellcome Trust Centre for Neuroimaging, University College London. Participants were initially briefed that they would be making choices about how to allocate relief from different numbers of moderately painful electric shocks. Throughout the experiment the participant sat in front of a computer monitor; where trials were presented on-screen, and decisions were indicated using keys on the keyboard.

Two participants were excluded prior to coding and analysis of data because at the end of the experimental run they stated that they did not find the painful stimulation aversive, creating a dataset of 33 participants (S1 Dataset). Three were excluded from the analysis, since they performed a pilot version of the task in which they did not receive on-screen information regarding the mean relief remaining per trial. The remaining 30 participants all also took part in the binary intertemporal choice experiment, which they performed first, on the same day as the relief consumption task (published previously). Anticipation-discounting parameters were estimable in 23 participants from these thirty. The remaining 7 participants always choose sooner pain on the binary choice experiment, precluding reliable model fitting [See 33].

**Procedure and design: dynamic task**. Participants made choices over an experimental session consisting of 60 trials in which by default they were due to receive painful shocks on each trial. Participants were briefed with on-screen instructions that embedded the task in a naturalistic health-related scenario (see S1 Text). At the start of the session participants were endowed with a fixed budget of computerized pain relief, described in units of milligrams, 2400mg in total. The budget was not sufficient to relieve all the shocks in the session, and participants were informed of this fact, and therefore the possibility that they might expend all their relief before the end of the session. Before each trial, participants were informed of the total number of trials remaining, the number of units of relief remaining and the calculated mean relief remaining per trial in mg. They were then given the opportunity to indicate how much relief they wished to consume on that trial, by moving a pointer along a visual scale using the keyboard. There followed a painful shock stimulus, the severity of which was determined by the amount of relief consumed.

The painful shocks occurred within a five second stimulus train, where the intensity of each discrete shock, which consisted of a single 200μs square-wave pulse, did not vary. The duration of the stimulus was fixed therefore an increasing number of shocks was equivalent to an increasing shock rate. At each sampled time interval during the stimulus train the probability of receiving a shock was sampled from a uniform distribution. By default the outcome on each trial was a shock train with the maximum rate of 2.8 shocks/s (14 shocks within 5 seconds). Consuming 10mg of relief reduced the expected number of shocks in the immediately following stimulus train by one. Participants were informed that the pain relief was probabilistic, chosen so as to achieve a more naturalistic context. The maximum allowable consumption of relief on each trial was 120mg, sufficient to reduce the expected shock rate to 2 shocks/5s (0.4 shocks/s), which was referred to as the “Baseline Pain”. Prior to entering into the session, participants were given three samples of the maximum (default) and minimum (baseline) shock rates which they could expect to experience with using no relief or using maximum relief respectively. The choice phase was limited to 6 seconds, and each trial lasted 14 seconds in total, the experimental session therefore lasted 14 minutes.

Before the experiment, participants underwent a standardized procedure, to control for individual variability in pain perception, so that the maximum shock rate used during the experiment corresponded to an approximately equivalent subjective level of pain for each participant. We aimed to set a target current level (the stimulator then adjusted the voltage to achieve this target current) such that participants rated the five second stimulus at the maximum shock rate (2.8 shocks/s) as moderately severe pain. To achieve this we used an expected shock rate of 2.8 shocks/s, whilst varying the target current amplitude. Participants provided a pain rating for each stimulus train on a continuous visual analogue scale (VAS) from 0 (not painful) to 10 (intolerable pain). Voltage level was increased in small increments until the participant gave the stimulus a VAS rating of 6 out of 10. The staircase procedure was then repeated, giving participants opportunity to adapt to initial anxiety about the shocks. This procedure determined a single voltage level corresponding to moderately severe pain for each participant. At the end of the experimental session we also verified that increasing the mean shock rate within the range used for the experiment corresponded to monotonic increases in VAS pain ratings, by asking participants to rate stimulus trains of constant voltage, equal to that used during the choice phase, whilst shock rate was increased in increments of 2 shocks/5s, starting from the baseline mean rate of 2 shocks/5s up to the maximum rate of 14 shocks/5s. This was followed by a symmetrical decreasing staircase in which shock rate was decreased by the same increment. 2 out of the 35 participants rated the maximum shock rate as below 4/10 (which corresponded to “mild pain” on the visual analog rating scale) at the end of experiment, suggesting that significant adaptation had occurred over the course of the experiment. These 2 participants were therefore excluded from the analysis.

**Procedure and design: one-off choices**. The procedure for estimating temporal value functions from one-off binary intertemporal choices has been described elsewhere [9]. In brief, the experiment proceeded according to a trial-based design in which the unit of time was a single trial and participants’ choices determined outcomes on future trials. The painful shocks were delivered within a five second stimulus train, identical to that used in the dynamic choice setting. Prior to making their choices participants received samples of stimulus trains at different shock rates, so that they were familiar with the outcomes. On each trial the default outcome was a shock train with mean 2 shocks/5s (0.4 shocks/s), identical to the “Baseline Pain” in the dynamic setting. Participants made two sets of 95 choices between two options for outcomes with higher expected shock rates, up to a maximum of 14 shocks/5s (i.e. 2.8 shocks/s, identical to the maximum rate in the dynamic context), delivered at between 4 to 51 trials in the future. There was an equal number of choices in which the delayed outcome had a higher expected shock rate as choices in which the sooner outcome had a higher expected shock rate. Each trial lasted an average of approximately 10 seconds in total, equivalent to the duration of a single trial in the dynamic setting. All choices were genuine, with shock delivered reliably according to subjects’ choices.

Participants were briefed with instructions that embedded the task in a health-related scenario, similar to that used for the dynamic choice setting. Intertemporal choice data was collected in two blocks, the order of which was counterbalanced: a block in which outcomes were framed as an increase in shock rate, referred to as the ‘pain’ frame and an otherwise identical experimental block in which outcomes were framed as a decrease in shock rate from the maximum rate, referred to as the ‘relief’ frame. The same participants performed these static intertemporal choices, prior to the dynamic choice experiment, on the same day. Responses were analyzed by fitting a series of alternative temporal value functions to participants’ choices using maximum-likelihood estimation. The best-fitting class of model was an exponential-sum dread model of the form described below.

**Data processing**. To reduce the computational complexity of the modeling and simulation analysis (necessary when fitting more complex models using dynamic programming), relief consumption was rounded to the nearest 10mg, creating 13 possible spending choices on each trial (0 to 12). This procedure produced occasional rounding errors such that the cumulative total rounded consumption exceeded the budget constraint. These errors were corrected by disallowing rounded consumption to exceed the remaining total relief, resulting in fictitious observations on the final trial for some participants. These discrepancies from the true observed consumption profiles were small by comparison to predominant patterns of consumption.

**Simulating consumption paths**. To simulate consumption paths predicted by the dread-discounting functions derived from one-off choices we implemented a dynamic program [54–56] over all possible states of capital at each time point. A deterministic transition function, *T*(*c_t_*, *s_t_*) described how actions in the current state mapped to subsequent states, such that:

$$s_{t+1}=s_{t}-c_{t}$$ 7)

Where *s_t_*, denotes capital at time *t*, and *c_t_* consumption at *t*. Borrowing is not allowed, therefore *s_t_* ≥0 and *c_t_*≤*s_t_*.

Consuming a quantity of relief, *c_t_*, was associated with utility *U*(*c_t_*) at the current state, where *U*(*c_t_*) is the utility function for relief. Since *c_t_* ≤*s_t_*, the function *U* also depends on current capital *s_t_*. The overall value of consuming relief, *c_t_*, when situated at time, *t*, with a state of capital, *s_t_*, termed a *Q*-*value*, was then described recursively as a function of the resulting relief utility at the current state, *U*(*c_t_*, *s_t_*), followed by the expected utility of relief at all future states, given a future action policy, π, and a discount function, Δ(*d*), giving rise to:

$$Q^{\pi }\left(s_{t},c_{t}\right)=U\left(c_{t},s_{t}\right)+E\left[\sum_{d-1}^{T-t}\Delta \left(d\right)\cdot U{\left(c_{t+d},s_{t+d}\right)}_{c_{t+d}\sim \pi }\right]$$ 8)

The action policy, π, dictates the probability of consuming an amount of relief, *c_t_*, given that the agent is currently situated at *t* and has capital, *s_t_*, here represented by a softmax policy for action selection, such that:

$$p\left(c_{t}\text{|}s_{t},t\right)=\pi \left(s_{t},t\right)$$ 9)

Where:

$$\pi :s_{t},t\to \frac{e^{\beta Q^{\pi }(s_{t},c_{t},t)}}{\sum_{c_{t}}e^{{}^{\beta Q^{\pi }(s_{t},c_{t},t)}}}$$ 10)

Higher values of the inverse temperature parameter, *β*, lead to behavior becoming more deterministic for choosing the option with higher utility. Δ(*d*) was represented by the anticipation-discounting function derived from one-choices, which assumed the following form:

$$\Delta \left(d\right)=\gamma_{C}{}^{d}+\alpha \left[\sum_{\tau =0}^{d-1}\gamma_{C}{}^{d-\tau }\gamma_{A}{}^{\tau }\right]$$ 11)

Parameters from the Exponential Dread model (*γ_D—_ framing*; both Pain and Relief frames separately), namely *α, γ_P_* and *γ_D_* were carried forward to generate resulting optimal consumption paths on the dynamic experiment. To do so, discounting of relief consumption was set to be equivalent to discounting of pain (*γ_C_* = *γ_P_*) and discounting of dread was set to be equivalent to the discounting of savoring (*γ_A_* = *γ_D_*). A linear utility function for pain and relief was assumed. Optimal policies were implemented using a high value of the softmax inverse temperature, *β* = 10000.

The value function *Q* ^π^(*s_t_*, *c_t_*) expresses the notion that the value of consuming an amount, *c*, at the current capital level and time period depends on the immediate utility of consuming *c* plus the expected value of (discounted) future consumption, given accurate knowledge of one’s likely future policy for action. This model therefore entails complete sophistication. In other words the model assumes for example that the agent at *t* = knows that a future decision-maker at *t* = 11 will apply the same degree of discounting to periods *t* = 11,12,13… and so on as the agent currently applies to periods *t* = 1,2,3… and so on. Naivety by contrast would entail for example that the agent at *t* = 1 assumes that the decision-maker at *t* = 11 will apply the same discount factors to periods *t* = 11,12,13… and so on as the agent currently applies to those time periods. Given dynamic inconsistency in the discounting function, a naïve agent would be expected to change their plans at each time step.

To explore the predicted future plans resulting from different forms of anticipation-discounting and utility functions, under naivety as well as sophistication, we also simulated the behavior of an agent facing a discrete-time dynamic intertemporal reward allocation task lasting 10 time periods. The agent was endowed with a budget of 100 units of reward at the start of the task, and was allowed to consume any proportion of the total remaining reward (capital) at each time period. There was no experimenter-determined interest rate on assets not yet consumed. To simulate naïve behavior, the form of the discount function was made dependent on the absolute timing of the outcomes as well as their delay, such that the decision-maker at each time step believed that future decision-makers would apply the same preferences as those currently held for those time-periods. To achieve this, the dynamic program was iterated once for each trial of the simulation, with the following recursive value function:

$$)Q_{naive}^{\pi }\left(s_{t},c_{t},t,i\right)=\Delta \left(t-i\right)\cdot U\left(c_{t},s_{t}\right)+E\left[\sum_{\tau =t+1}^{T}\Delta \left(\tau -i\right)\cdot U{\left(c_{t+d},s_{t+d}\right)}_{c_{t+d}\sim \pi }\right]$$ 12)

Where each iteration is represented by *i*, which ranges between 1 and *T*. Naive consumption plans (as shown in Fig. 4C) at trial *i* were sampled from a policy based on $Q_{naive}^{\pi }\left(s_{t},c_{t},i\right)$ over the remaining trials *t* = *i*, *i*+*1*, *i*+*2*… *T*. Naïve consumption paths themselves were simulated by sampling from $Q_{naive}^{\pi }\left(s_{t},c_{t},t\right)$ over all trials *t* = 1,2,3… *T*.

**Model fitting procedures**. For each of the models, we assumed a standard probabilistic model of action selection in the form of a softmax function. Model fitting followed a maximum likelihood framework, using the softmax policy to generate the probability of observing each possible (rounded) level of relief consumption, given a particular set of model parameters. For each model we sought parameters which maximized the log likelihood of (minimized the negative log likelihood) of the observed consumption choices of each participant. To do so, simplex optimization was performed using the Matlab (Mathworks, MA, USA) fminsearch optimization tool (Nelder-Mead search algorithm [57]) with the addition of bound constraints by transformation. For each subject 10 iterations of the optimization were performed, and the maximum likelihood estimate across all iterations was selected. On each iteration the optimizer was called within a random multi-started overlay (RMsearch), with 100 starting points selected from a uniform distribution between the parameter bounds, in order to reduce convergence on local minima.

To find the best-fitting values of the softmax temperature parameter, *β*, and the exponent of the utility function, *k*, assuming that participants behaved so as to maximize the previously-derived anticipation-discounting functions, we implemented the value function shown in Equation 8, assuming full sophistication, whilst optimizing over *β* and *k*.

The value function of the Direct Action heuristic model is described in the main text. The Direct Action model had only three parameters: the inverse temperatures of each softmax function, respectively termed, *β_spend_*, *β_spread_* and *β_save_*. The value function for the Income Maximization model was identical to the Direct Action model, with the exception that the propensity to spend-now-save-later, *M_save_*, was replaced with an action-value function, the maximization of which maximizes the mean relief remaining, *ρ_t_*, over the immediate future, given knowledge of the future policy for action, *π*:

$$Q_{\rho -max}^{\pi }\left(s_{t},c_{t},t,\gamma \right)=\rho_{t}\left(s_{t},t\right)+E\left[\sum_{d=1}^{T-t}\gamma^{d}\rho_{t+d}{\left(s_{t+d,}t+d\right)}_{c_{t+d}\sim \pi }\right]$$ 13)

Where:

$$\rho_{t}\left(s_{t},t\right)=s_{t}/\left[60–\left(t-1\right)\right]$$ 14)

The Income Maximization model thus had four parameters: *β_spend_*, *β_sread_*, *β_maximize_* and *γ*, where the latter governs the probability, *1*-*γ*, that the decision-maker terminates their search at every level deeper into the tree. For both heuristic models, the softmax temperature of each policy was bounded between 0 and 10. The parameter, *γ*, governing the search depth of the Income Maximization model, was bounded between 0 and 1. For these models, the resulting three policies were combined by a weighted average, in which the weight given to each policy was proportional to the inverse variance of the resulting distribution of consumption choices, such that:

$$\omega_{i}=\left[\frac{1}{var\left(\pi_{i}\right)}\right]/\overset{3}{\underset{j=1}{\sum }}\left[\frac{1}{var\left(\pi_{j}\right)}\right]$$ 15)

Where *ω_i_* is the weighting on policy, *π_i_*. This procedure served as a useful heuristic for combining policy estimates.

To combine heuristics with utility maximization, we assumed that the value of consuming each possible quantity of relief at each time point was governed by a weighted sum of the value function in Equation 8, here termed $Q_{opt}^{\pi }\left(s_{t},c_{t}\right)$ and a bias towards consuming either zero, the mean remaining or the maximum relief. Each bias assumed that the propensity to spend each possible quantity of relief was proportional to a Gaussian probability density function with a mean centered on the quantity of interest, and standard deviation equal to two units of relief, such that:

$$M_{min}\sim N\left(0,2\right)$$ 16)

$$M_{mean}\left(\rho_{t}\right)\sim N\left(\rho_{t},2\right)$$ 17)

$$M_{max}\left(s_{t}\right)\sim \{\begin{array}{c} N\left(12,2\right)ifs_{t}\geq 12\\ N\left(s_{t},2\right)otherwise \end{array}$$ 18)

The final value function, $Q_{opt-heur}^{\pi }\left(s_{t},c_{t}\right),$ was then a weighted sum of the optimal values and the biases:

$$Q_{opt-heur}^{\pi }\left(s_{t},c_{t}\right)=Q_{opt}^{\pi }\left(s_{t},c_{t}\right)+\omega_{min}M_{min}+\omega_{mean}M_{mean}\left(\rho_{t}\right)+\omega_{max}M_{max}\left(s_{t}\right)$$ 19)

Fixed effects model comparison was performed at the group level by summation of log likelihoods across participants. Model comparison used the Bayesian Information Criterion (*BIC*) [58], where

BIC=−2L+kln(n) 20)

and *L* is the maximized group level log likelihood, *k* is the number of free parameters in the model and *n* the number of independent observations. The *BIC* favors models with higher likelihood estimates and penalizes increasing model complexity. Lower values of *BIC* indicate a more favorable model fit.

## Supporting Information

S1 Fig Dynamically inconsistent savoring. Anticipation-discounting functions of the form displayed in Fig. 3. **A** Where prospective savoring dominates, preference reverses towards deferral of consumption. Here r_1_ is a larger sooner reward and r_2_ is a smaller later reward, where both generate a large degree of savoring. When both rewards are distant, the larger, sooner reward is preferred, however as the rewards approach, prospective savoring from both rewards diminishes at an increasing rate, such that the smaller delayed reward becomes preferable. **B** Where discounting dominates, preference can reverse towards sooner consumption, in a similar manner to conventional hyperbolic discounting. Here r_1_ is a smaller sooner reward and r_2_ is a larger later reward, where both generate a small degree of savoring. Here, in the absence of savoring the sooner reward, r_1_, would be preferred, due to exponential discounting. With savoring however, when both rewards are distant, the larger, later reward is preferred, due to its relatively greater savoring. Only as the sooner reward approaches in time, and its value increases due to decreased discounting, does it become preferable. The parameters of the functions are displayed on each plot.
(TIF)

S2 Fig Histograms of relief consumption over time. **A** Relief consumption expressed as mg. Each plot represents the distribution of relief consumption over a period of 10 trials. It is evident that multiples of 10mg are over-represented, consistent with a round-number heuristic. **B** Relief consumption rounded to the nearest 10mg, expressed as ‘units’. Each plot represents the distribution of relief consumption over a period of 10 trials. The data suggest save-now-spend-later and spread-spending heuristics.
(TIF)

S3 FigRelief consumption across participants exhibiting “spreading” of consumption.For these 13 participants the mean absolute deviation from even consumption, $\bar{\left|d_{t}\right|}$, was less than 1 unit of relief, an arbitrary threshold. Participants are arranged in ascending order of the variance of |*d_t_*|, which indicates the trial-to-trial deviation from even utility spreading. **A:** The first six participants adhere relatively closely to even utility spreading on a trial-to-trial basis. The next five participants maintain even utility spreading when averaged across trials, but show a greater degree of variability in their choices on a trial-to-trial basis. **B:** The final two participants, whilst spreading consumption over time, appear to demonstrate mixed profiles of relief consumption, including a tendency to spend close to either the maximum (12 units) or minimum (0 units) allowable quota of relief.(TIF)

S4 FigRelief consumption across participants exhibiting “saving”.The 15 participants for whom $\bar{d_{t}}\leq -1$. It is evident that some participants chose nearly exclusively to conserve relief until the mean relief remaining, *ρ_t_*, reached the maximum allowable spend per trial of 12 units. However, several participants appeared to employ mixed policies for consumption.(TIF)

S5 FigRelief consumption across participants classified as exhibiting “early spending”.The 2 participants for whom $\bar{d_{t}}\geq +1$. Trial-to-trial consumption is highly variable, rather than reflecting a deterministic policy to spend the maximum allowable relief, suggesting that these participants may have chosen consumption almost randomly for the majority of the experimental run. As *ρ_t_* declines, both participants make attempts to constrain their spending in line with this decline.(TIF)

S6 Fig Participant-level fits of the income maximization model. **A** Observed rounded relief consumption profiles (blue circles) for the six participants whose data is displayed in Fig. 5, overlaid with consumption simulated (red circles) from the maximum likelihood parameterization, *θ*, of the Income Maximization model. Whilst the model fitting process takes account of the observed state of capital on each trial, the simulated paths here are sampled anew from the maximum likelihood parameterization without reference to the data. **B** Color plots indicating probability across all participants of consuming an amount of relief, *c_t_*, on each trial, *t*, given a vector of the total remaining relief for each participant on each trial, *s_t_*, *s_t_*_+1_, *s_t_*_+2_, … s_T_, at the maximum likelihood parameterization, *θ*, of each model overlaid with observed consumption data (white circles). It is evident that the model is able to account for the main behavioral tendencies, as well as their dynamics.
(TIF)

S7 Fig Combining anticipation-discounting with heuristics. **A** The observed distribution of consumption by all 30 participants included in the analysis. Warmer colors indicate that a higher proportion of participants chose to consume that amount of relief on a particular trial. **B** Group-Level distribution of relief consumption predicted by anticipation-discounting functions derived from relief frame choices, with the softmax temperature, beta, and utility parameters freely fitted, with a varying degree of bias towards consuming either the minimum, maximum or mean remaining relief on each trial. The plot denotes the mean probability across all participants of consuming an amount of relief, *c_t_*, on each trial, *t*, given a vector of the total remaining relief for each participant on each trial, *s_t_*, *s_t_*_+1_, *s_t_*_+2_, … s_T_, at the maximum likelihood parameters, *θ*, of each model. **C** The proportion of variance explained by the model. Mean predicted consumption levels simulated from the maximum likelihood parameterizations over each 10 trials of the experiment for each participant are plotted against the same metric derived from the observed data.
(TIF)

S1 Text Information given to participants. This text file details the on-screen instructions used to brief participants in the relief scheduling experiment.
(DOCX)

S1 Table Model parameters for the Income Maximization Heuristic Model. The maximum likelihood parameter estimates for each participant from fits of the Income Maximization model. Parameters *β_save_*, *β_spend_* and *β_spread_* are the softmax inverse temperatures on the three behavioral tendencies, to maximize the mean relief remaining, to spend the maximum allowable relief and to spend close to the mean relief remaining respectively. The *γ* parameter denotes the probability of searching one step deeper into the decision tree at each stage whilst attempting to maximize the mean remaining relief, akin to exponential discounting of future wealth.
(DOCX)

S1 Dataset Relief consumption profiles. Contains the raw data for relief consumption in milligrams by each of the 33 participants who rated the shocks as aversive, on each of 60 trials. The three subjects highlighted in gray were excluded from the analysis since they were not provided with on-screen details of the mean relief remaining per trial.
(XLSX)

S2 DatasetBinary intertemporal choice metrics and dread-discounting parameters.Data summarizing behavior on the previously published binary intertemporal choice task; the frequency with which each participant chose the sooner of the two options for delayed painful shocks in binary choice experiment (previously published, [33], S1 Table), and the maximum likelihood parameter estimates resulting from fitting an exponential dread-discounting model (previously published, [33], *γ_P_—framing* model) to the observed choices. This file also contains the derived metric, $\bar{d},$ used to classify behavior on the dynamic consumption experiment.(XLSX)
