# Supplementary figures and images for: Anticipation and Choice Heuristics in the Dynamic Consumption of Pain Relief
Source: PLoS Comput Biol. 2015 Mar 20;11(3):e1004030. doi: 10.1371/journal.pcbi.1004030 (PMC4368544; doi:10.1371/journal.pcbi.1004030)

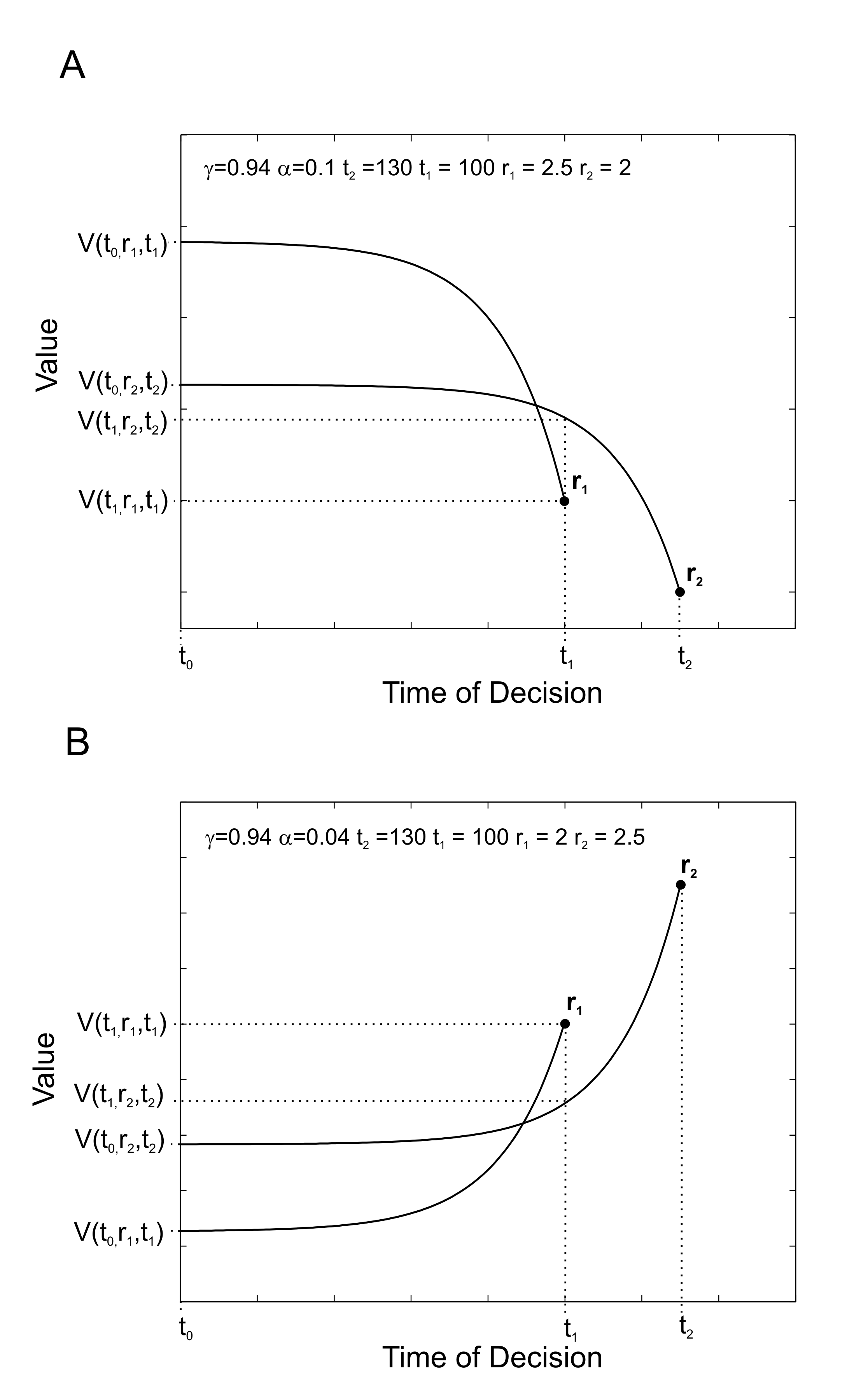

Supplement: S1 Fig — Anticipation-discounting functions of the form displayed in Fig. 3. A Where prospective savoring dominates, preference reverses towards deferral of consumption. Here r1 is a larger sooner reward and r2 is a smaller later reward, where both generate a large degree of savoring. When both rewards are distant, the larger, sooner reward is preferred, however as the rewards approach, prospective savoring from both rewards diminishes at an increasing rate, such that the smaller delayed reward becomes preferable. B Where discounting dominates, preference can reverse towards sooner consumption, in a similar manner to conventional hyperbolic discounting. Here r1 is a smaller sooner reward and r2 is a larger later reward, where both generate a small degree of savoring. Here, in the absence of savoring the sooner reward, r1, would be preferred, due to exponential discounting. With savoring however, when both rewards are distant, the larger, later reward is preferred, due to its relatively greater savoring. Only as the sooner reward approaches in time, and its value increases due to decreased discounting, does it become preferable. The parameters of the functions are displayed on each plot. (TIF) [file pcbi.1004030.s001.tif]

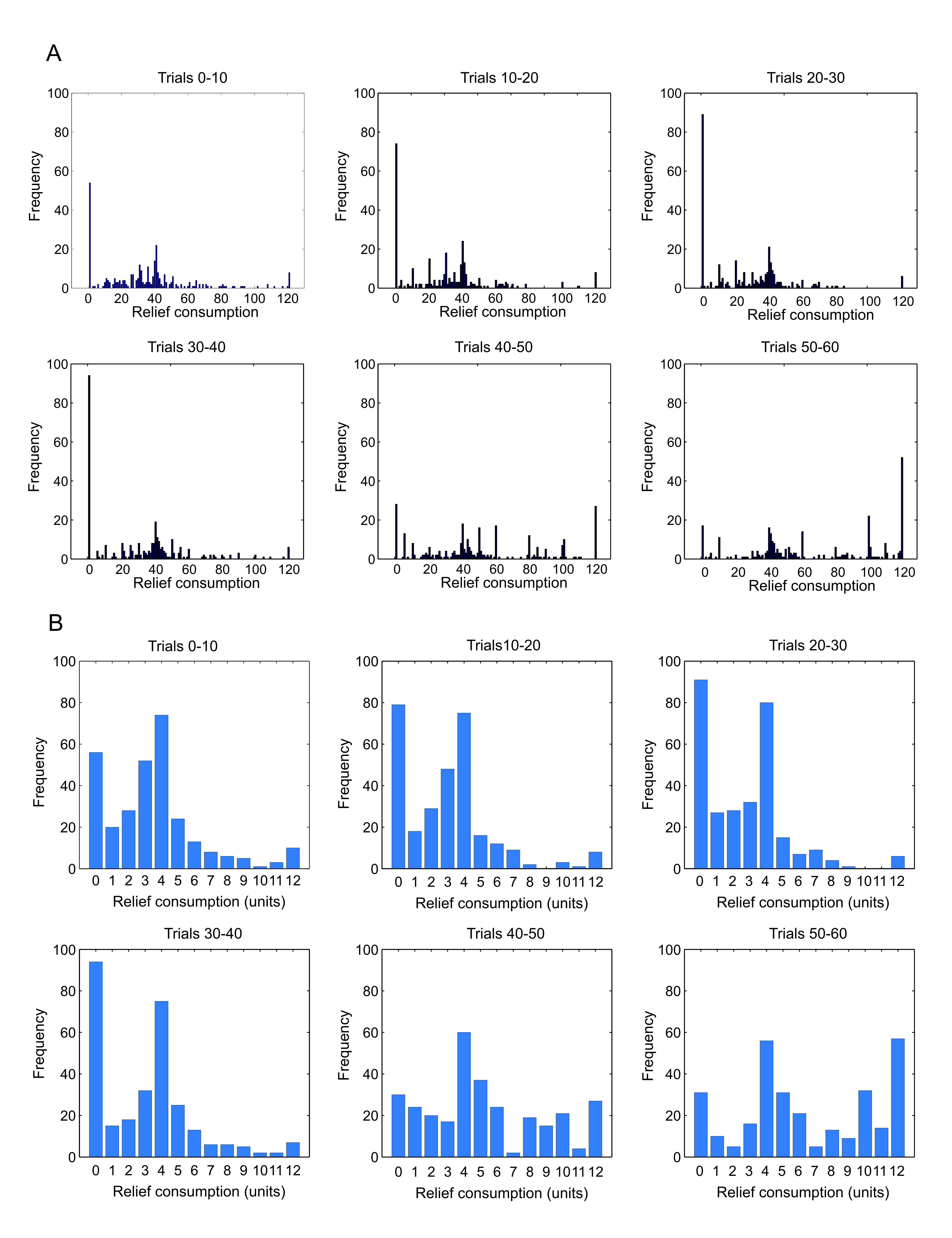

Supplement: S2 Fig — A Relief consumption expressed as mg. Each plot represents the distribution of relief consumption over a period of 10 trials. It is evident that multiples of 10mg are over-represented, consistent with a round-number heuristic. B Relief consumption rounded to the nearest 10mg, expressed as ‘units’. Each plot represents the distribution of relief consumption over a period of 10 trials. The data suggest save-now-spend-later and spread-spending heuristics. (TIF) [file pcbi.1004030.s002.tif]

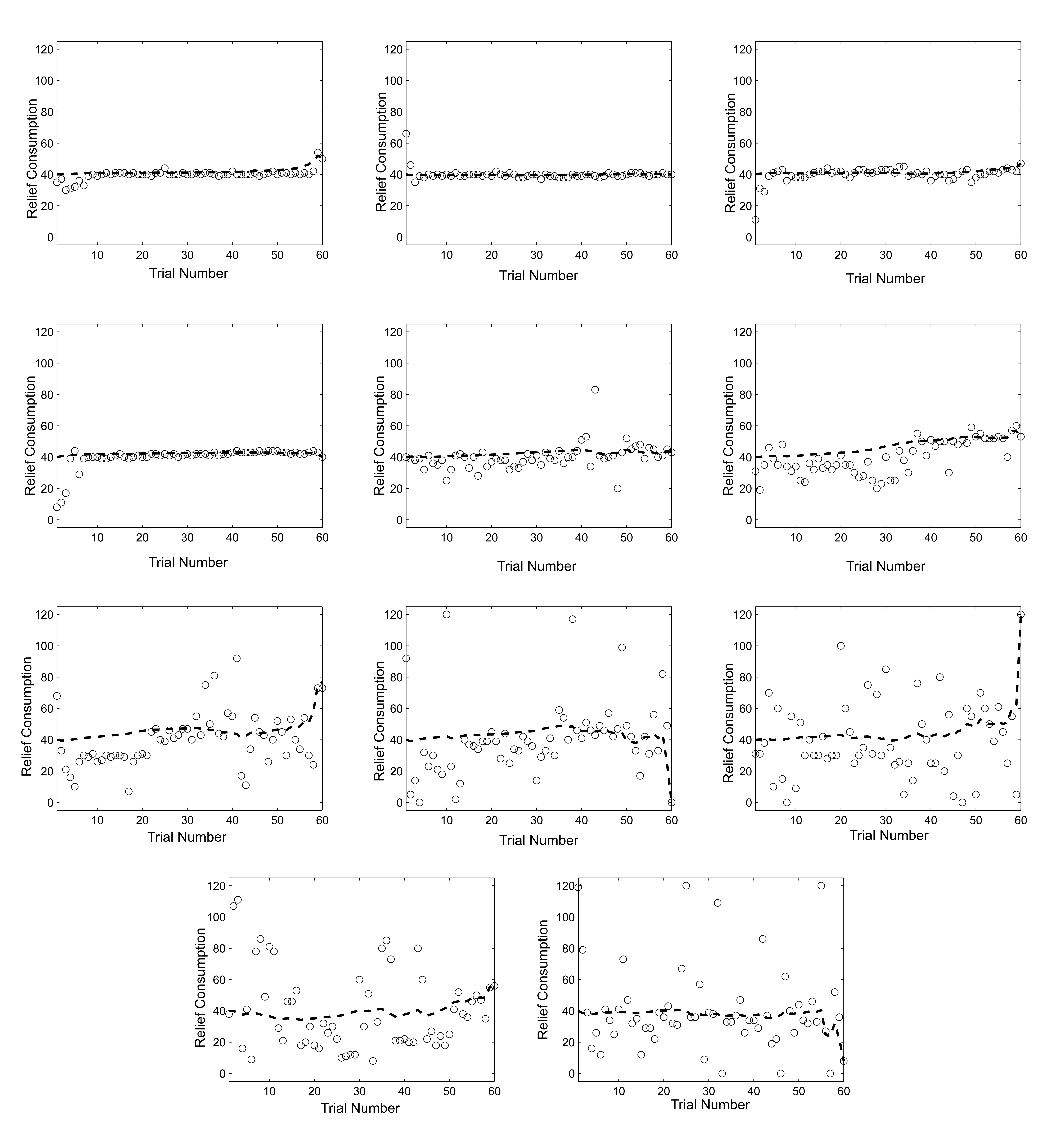

Supplement: S3 Fig — For these 13 participants the mean absolute deviation from even consumption, |dt|¯, was less than 1 unit of relief, an arbitrary threshold. Participants are arranged in ascending order of the variance of |dt|, which indicates the trial-to-trial deviation from even utility spreading. A: The first six participants adhere relatively closely to even utility spreading on a trial-to-trial basis. The next five participants maintain even utility spreading when averaged across trials, but show a greater degree of variability in their choices on a trial-to-trial basis. B: The final two participants, whilst spreading consumption over time, appear to demonstrate mixed profiles of relief consumption, including a tendency to spend close to either the maximum (12 units) or minimum (0 units) allowable quota of relief. (TIF) [file pcbi.1004030.s003.tif]

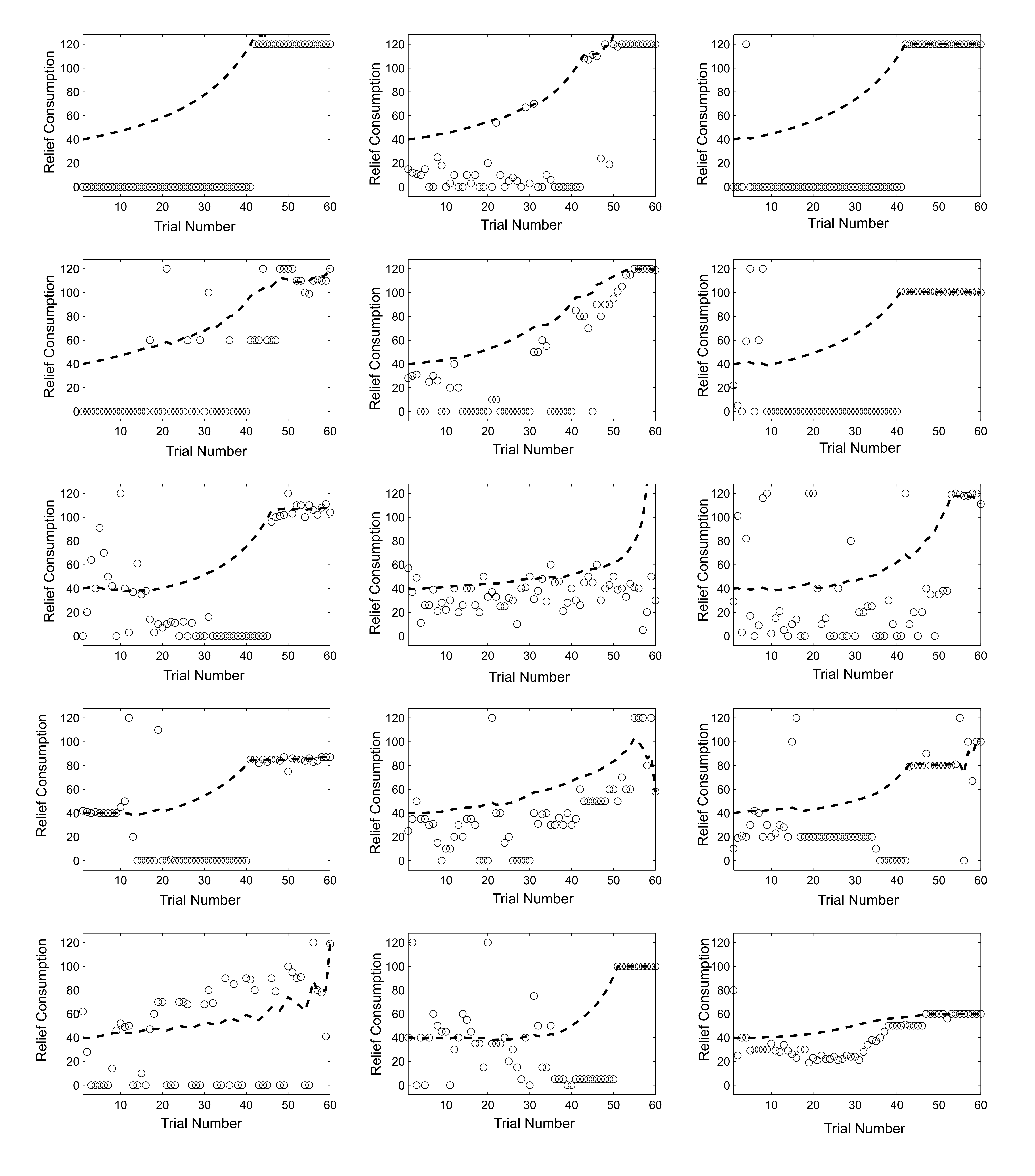

Supplement: S4 Fig — The 15 participants for whom dt¯≤−1. It is evident that some participants chose nearly exclusively to conserve relief until the mean relief remaining, ρt, reached the maximum allowable spend per trial of 12 units. However, several participants appeared to employ mixed policies for consumption. (TIF) [file pcbi.1004030.s004.tif]

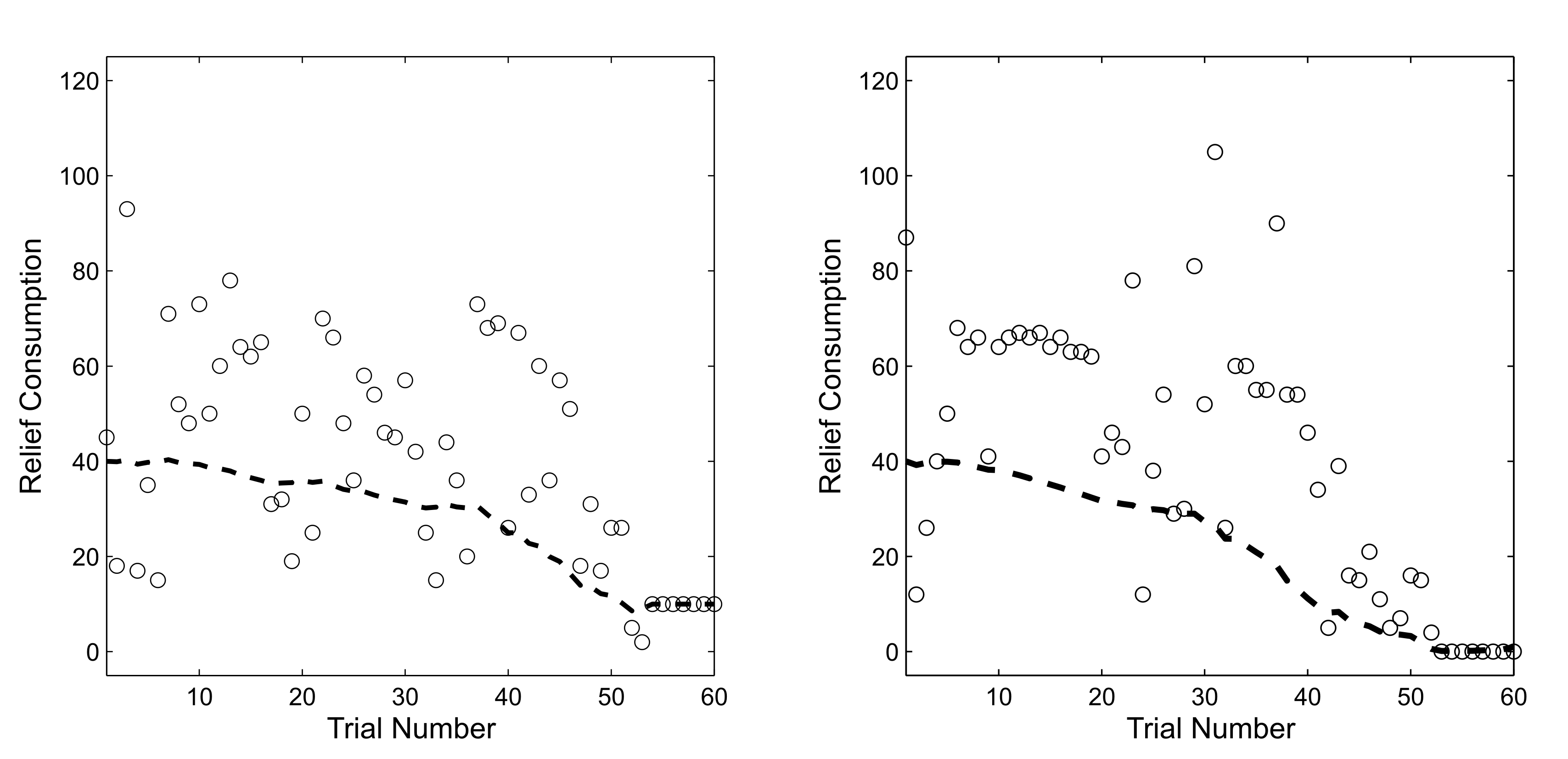

Supplement: S5 Fig — The 2 participants for whom dt¯≥+1. Trial-to-trial consumption is highly variable, rather than reflecting a deterministic policy to spend the maximum allowable relief, suggesting that these participants may have chosen consumption almost randomly for the majority of the experimental run. As ρt declines, both participants make attempts to constrain their spending in line with this decline. (TIF) [file pcbi.1004030.s005.tif]

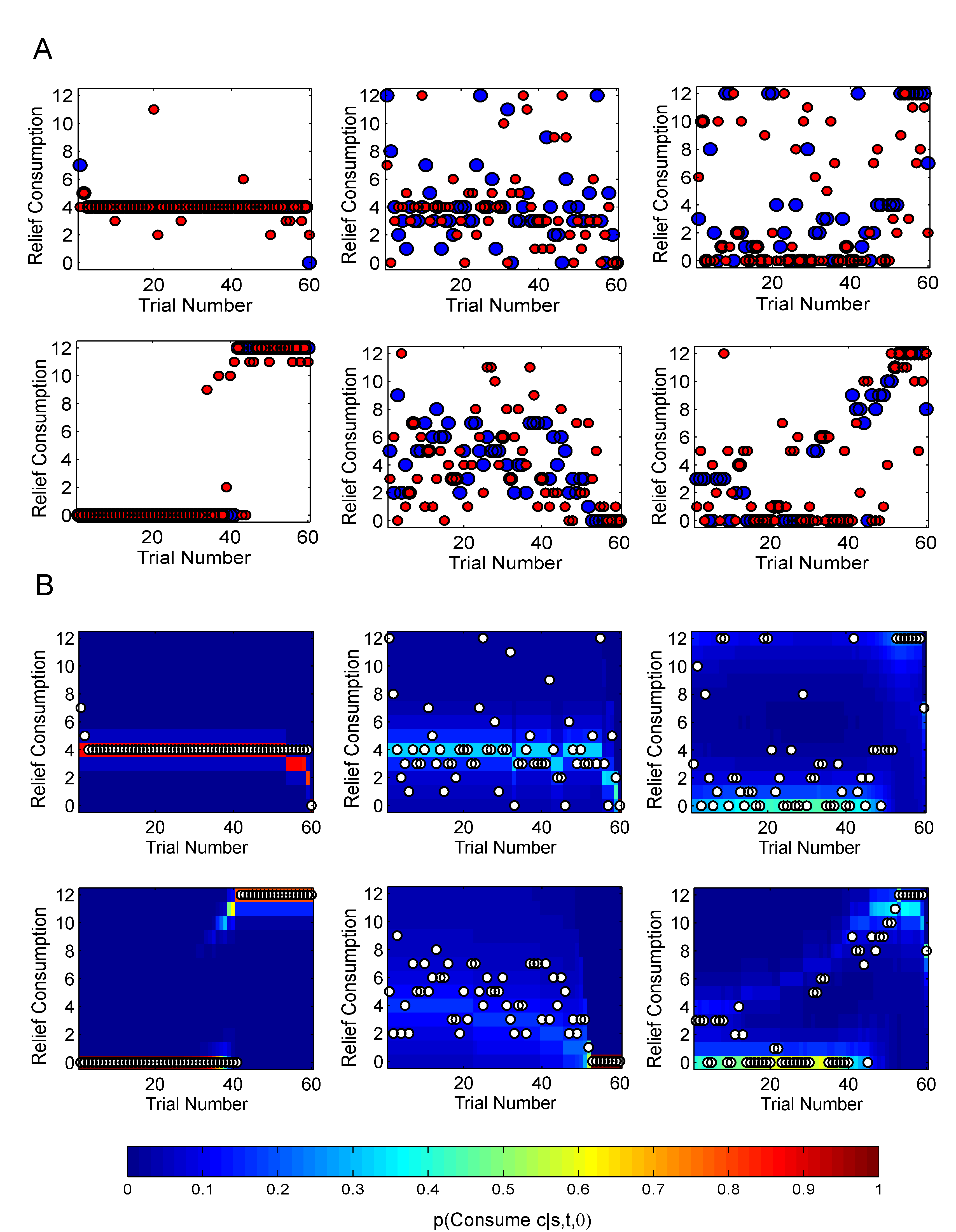

Supplement: S6 Fig — A Observed rounded relief consumption profiles (blue circles) for the six participants whose data is displayed in Fig. 5, overlaid with consumption simulated (red circles) from the maximum likelihood parameterization, θ, of the Income Maximization model. Whilst the model fitting process takes account of the observed state of capital on each trial, the simulated paths here are sampled anew from the maximum likelihood parameterization without reference to the data. B Color plots indicating probability across all participants of consuming an amount of relief, ct, on each trial, t, given a vector of the total remaining relief for each participant on each trial, st, st +1, st +2, … sT, at the maximum likelihood parameterization, θ, of each model overlaid with observed consumption data (white circles). It is evident that the model is able to account for the main behavioral tendencies, as well as their dynamics. (TIF) [file pcbi.1004030.s006.tif]

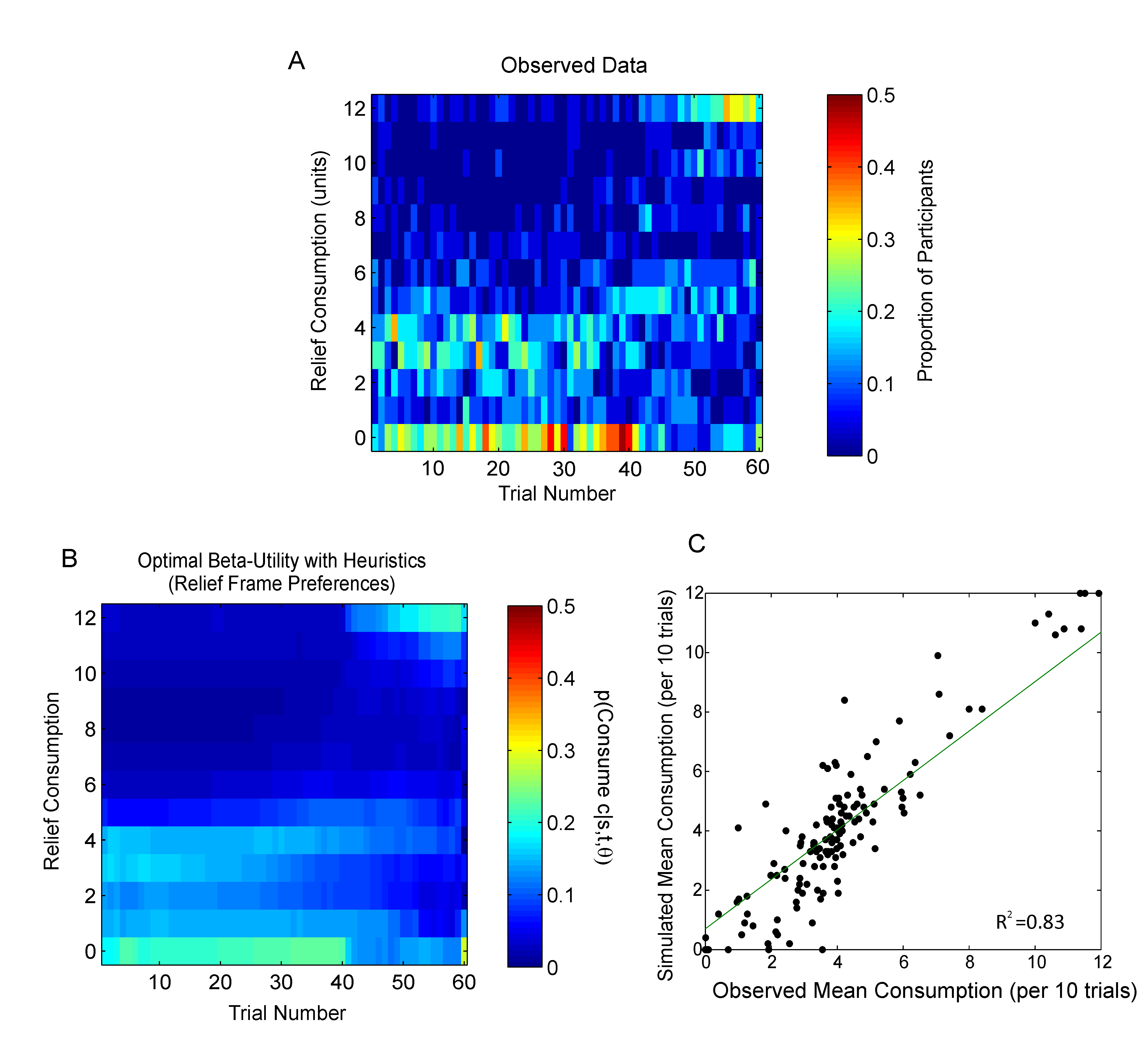

Supplement: S7 Fig — A The observed distribution of consumption by all 30 participants included in the analysis. Warmer colors indicate that a higher proportion of participants chose to consume that amount of relief on a particular trial. B Group-Level distribution of relief consumption predicted by anticipation-discounting functions derived from relief frame choices, with the softmax temperature, beta, and utility parameters freely fitted, with a varying degree of bias towards consuming either the minimum, maximum or mean remaining relief on each trial. The plot denotes the mean probability across all participants of consuming an amount of relief, ct, on each trial, t, given a vector of the total remaining relief for each participant on each trial, st, st +1, st +2, … sT, at the maximum likelihood parameters, θ, of each model. C The proportion of variance explained by the model. Mean predicted consumption levels simulated from the maximum likelihood parameterizations over each 10 trials of the experiment for each participant are plotted against the same metric derived from the observed data. (TIF) [file pcbi.1004030.s007.tif]
