# Supplementary material for: Anticipation and Choice Heuristics in the Dynamic Consumption of Pain Relief
Source: PLoS Comput Biol. 2015 Mar 20;11(3):e1004030. doi: 10.1371/journal.pcbi.1004030 (PMC4368544; doi:10.1371/journal.pcbi.1004030)
Supplement: S1 Text — This text file details the on-screen instructions used to brief participants in the relief scheduling experiment. (DOCX) [file pcbi.1004030.s008.docx]

Supporting Text 1. **Analysis of adaptation and mid-saving behavior**.

We are grateful to a reviewer for raising the interesting hypothesis that the tendency to save pain relief in the middle of the experiment might be related to adaptation over the course of the experiment. We are underpowered to detect all but the strongest of effects here, given the relatively small numbers of subjects displaying any given pattern of consumption. Nevertheless, we performed some preliminary analyses to test this, finding no relationship with the extent of adaptation.

The consumption data from subjects with a tendency to save in the middle of the experiment (‘mid-savers’) tend to be better explained by the *Income Maximization* model than by the *Direct Action* model. We therefore regressed the log likelihood ratio between the two models against the degree of adaptation over the course of the experiment, expressed as a change in VAS score for a stimulus with the maximum shock rate. We found no significant correlation (*N* = 30, Pearson *r* = -0.09, p = 0.63).

More subjectively we also classified ‘mid-savers’ simply by visual inspection of the consumption profiles – identifying seven subjects who showed this pattern (Figure S2B [1,2], Figure S3 [2,2], [3,1], [4,1], [4,2], [4,3] and [5,2]; where the numbers in square brackets indicate the row and column for each subject’s data). For the seven ‘mid-savers’, the median change in VAS score over the course of the experiment was -0.18, and for the remaining 23 participants the median change was 0.38, indicating a trend towards sensitization in the mid-savers, opposite to the expected direction. However the difference was not significant (Wilcoxon rank sum test, p = 0.20).
