# Supplementary material for: Anticipation and Choice Heuristics in the Dynamic Consumption of Pain Relief
Source: PLoS Comput Biol. 2015 Mar 20;11(3):e1004030. doi: 10.1371/journal.pcbi.1004030 (PMC4368544; doi:10.1371/journal.pcbi.1004030)
Supplement: S1 Table — The maximum likelihood parameter estimates for each participant from fits of the Income Maximization model. Parameters βsave, βspend and βspread are the softmax inverse temperatures on the three behavioral tendencies, to maximize the mean relief remaining, to spend the maximum allowable relief and to spend close to the mean relief remaining respectively. The γ parameter denotes the probability of searching one step deeper into the decision tree at each stage whilst attempting to maximize the mean remaining relief, akin to exponential discounting of future wealth. (DOCX) [file pcbi.1004030.s009.docx]

Table S1. **Model parameters for the *Income Maximization* Model**

| Participant | * β_save_* | * β_spend_* | * β_spread_* | γ |
| --- | --- | --- | --- | --- |
|  |  |  |  |  |
| 1 | 2.112 | 0.000 | 2.378 | 0.240 |
| 2 | 0.316 | 0.000 | 2.405 | 0.639 |
| 3 | 1.215 | 0.468 | 0.440 | 0.944 |
| 4 | 1.629 | 0.454 | 0.049 | 0.950 |
| 5 | 0.068 | 0.000 | 0.900 | 1.000 |
| 6 | 1.520 | 0.735 | 0.722 | 0.978 |
| 7 | 10.000 | 0.000 | 1.717 | 1.000 |
| 8 | 10.000 | 0.000 | 0.637 | 0.048 |
| 9 | 3.748 | 0.535 | 1.895 | 0.952 |
| 10 | 10.000 | 0.768 | 2.282 | 0.927 |
| 11 | 0.053 | 0.000 | 1.307 | 1.000 |
| 12 | 10.000 | 0.000 | 1.113 | 0.843 |
| 13 | 0.095 | 0.187 | 0.885 | 1.000 |
| 14 | 1.572 | 0.141 | 0.831 | 0.938 |
| 15 | 0.104 | 0.000 | 0.531 | 0.985 |
| 16 | 10.000 | 0.000 | 0.776 | 0.192 |
| 17 | 1.485 | 0.276 | 0.769 | 0.917 |
| 18 | 0.195 | 0.000 | 1.016 | 1.000 |
| 19 | 0.005 | 0.000 | 1.909 | 0.954 |
| 20 | 10.000 | 1.691 | 0.000 | 0.965 |
| 21 | 10.000 | 0.346 | 1.130 | 0.812 |
| 22 | 0.843 | 0.253 | 0.598 | 0.996 |
| 23 | 4.687 | 0.165 | 0.841 | 0.907 |
| 24 | 0.217 | 0.145 | 0.949 | 1.000 |
| 25 | 3.108 | 0.530 | 0.773 | 1.000 |
| 26 | 0.362 | 0.000 | 0.409 | 1.000 |
| 27 | 10.000 | 0.000 | 0.433 | 0.114 |
| 28 | 0.000 | 0.152 | 2.786 | 0.995 |
| 29 | 10.000 | 0.000 | 1.604 | 0.124 |
| 30 | 10.000 | 0.000 | 0.294 | 0.106 |
